# Monocytic Differentiation of Human Acute Myeloid Leukemia Cells: A Proteomic and Phosphoproteomic Comparison of FAB-M4/M5 Patients with and without Nucleophosmin 1 Mutations

**DOI:** 10.3390/ijms25105080

**Published:** 2024-05-07

**Authors:** Frode Selheim, Elise Aasebø, Håkon Reikvam, Øystein Bruserud, Maria Hernandez-Valladares

**Affiliations:** 1 Proteomics Unit of University of Bergen (PROBE), University of Bergen, Jonas Lies vei 91, 5009 Bergen, Norway; 2 Acute Leukemia Research Group, Department of Clinical Science, University of Bergen, Jonas Lies vei 91, 5009 Bergen, Norway; elise.aasebo@helse-bergen.no (E.A.); hakon.reikvam@uib.no (H.R.); oystein.bruserud@helse-bergen.no (Ø.B.); 3 Section for Hematology, Department of Medicine, Haukeland University Hospital, 5009 Bergen, Norway; 4 Department of Physical Chemistry, University of Granada, Avenida de la Fuente Nueva S/N, 18071 Granada, Spain; 5 Instituto de Investigación Biosanitaria ibs.GRANADA, 18012 Granada, Spain

**Keywords:** acute myeloid leukemia, Nucleophosmin 1 mutations, differentiation, monocytic, FAB classification, endocytosis, transcription

## Abstract

Even though morphological signs of differentiation have a minimal impact on survival after intensive cytotoxic therapy for acute myeloid leukemia (AML), monocytic AML cell differentiation (i.e., classified as French/American/British (FAB) subtypes M4/M5) is associated with a different responsiveness both to Bcl-2 inhibition (decreased responsiveness) and possibly also bromodomain inhibition (increased responsiveness). FAB-M4/M5 patients are heterogeneous with regard to genetic abnormalities, even though monocytic differentiation is common for patients with Nucleophosmin 1 (*NPM1*) insertions/mutations; to further study the heterogeneity of FAB-M4/M5 patients we did a proteomic and phosphoproteomic comparison of FAB-M4/M5 patients with (*n* = 13) and without (*n* = 12) *NPM1* mutations. The proteomic profile of *NPM1*-mutated FAB-M4/M5 patients was characterized by increased levels of proteins involved in the regulation of endocytosis/vesicle trafficking/organellar communication. In contrast, AML cells without *NPM1* mutations were characterized by increased levels of several proteins involved in the regulation of cytoplasmic translation, including a large number of ribosomal proteins. The phosphoproteomic differences between the two groups were less extensive but reflected similar differences. To conclude, even though FAB classification/monocytic differentiation are associated with differences in responsiveness to new targeted therapies (e.g., Bcl-2 inhibition), our results shows that FAB-M4/M5 patients are heterogeneous with regard to important biological characteristics of the leukemic cells.

## 1. Introduction

Acute myeloid leukemia (AML) is an aggressive malignancy characterized by proliferation of immature leukemia cells in the bone marrow [1,2]. Relatively young and fit patients can be cured by intensive chemotherapy, possibly combined with allogeneic stem cell transplantation, although only approximately half of the patients receiving this treatment achieve long-term AML-free survival [3,4]. Furthermore, elderly and unfit patients cannot receive such intensive therapy due to unacceptable risk of severe toxicity/treatment-related mortality, and many of these patients receiving only supportive or disease-stabilizing therapy (including targeted therapies) survive for less than half a year after diagnosis [2,3,4]. Thus, there is a need for new therapeutic strategies to improve antileukemic treatment both for young and elderly/unfit AML patients.

AML is a heterogeneous disease with regard to leukemia-associated genetic abnormalities, and one of the most common abnormalities is insertions in the *NPM1* gene (referred to as *NPM1-Ins*) [1,5,6]. A large subset of *NPM-Ins* patients show morphological signs of monocytic AML cell differentiation as defined by the French–American–British (FAB) and WHO 2016 M4/M5 classifications [7]. However, FAB-M4/M5 patients are heterogeneous with regard to genetic abnormalities and, thereby, also with regard to prognosis after intensive therapy; this includes genetic abnormalities associated with favorable/intermediate prognosis (e.g., *NPM1-Ins*) but also abnormalities associated with adverse prognosis [7,8,9,10,11,12]. Furthermore, the prognostic impact of *NPM1-Ins* seems to be relatively strong and can be detected even for patients with *NPM1-Ins* positive secondary AML. This was observed in a recent study outlining that patients with therapy-related *NPM1-Ins* AML had a long-term leukemia-free survival, similar to patients with de novo *NPM1-Ins* AML, whereas other secondary AML patients had decreased survival [13]. 

Several new drugs directed against specific molecular mechanisms are now considered for the treatment of AML [14], and many of these agents seem to induce differentiation of the AML cells [15]. Furthermore, although the morphological signs of AML cell differentiation have only very weak or no prognostic impact for AML patients receiving conventional intensive treatment [10,16,17,18], the responsiveness to some of the new targeted therapies is associated with monocytic differentiation. This is true for the Bcl-2 inhibitor venetoclax that is used in routine treatment in combination with azacitidine [19,20,21,22,23,24]; this treatment is associated with decreased responsiveness in monocytic and possibly also erythroid/megakaryocytic AML [19,21,22,23]. Experimental studies suggest that monocytic differentiation is also associated with resistance to CDK4/6 inhibitors [23], whereas monocytic AML shows relatively high responsiveness to bromodomain inhibitors [23]. As stated above, AML patients classified as monocytic FAB-M4/M5 AML are a heterogeneous group with regard to their genetic abnormalities and thereby also prognosis after intensive conventional chemotherapy, but *NPM1-Ins* patients then constitute a subset that probably share specific biological characteristics with relatively strong favorable prognostic impact. The aim of the present study was, therefore, to further investigate the heterogeneity of AML cells having monocytic differentiation as a common biological characteristic. Our study has a focus on protein networks/biological functions rather than individual proteins, and we compared the two biologically and clinically distinct subsets of monocytic AML cells with and without *NPM1-Ins*. 

## 2. Results

### 2.1. Characterization of FAB-M4/M5 Patients with and without NPM1 Insertions (-Ins)

We investigated 13 patients with and 12 patients without *NPM1*-*Ins.* These patients represent all FAB-M4/M5 patients from a consecutive group of relatively young patients fit for conventional intensive induction and consolidation therapy [25]. The clinical and biological characteristics of the patient subsets with and without *NPM1-Ins* are summarized in Table 1, and the detailed characteristics for each individual patient are presented in Table S1.

- The two groups did not differ significantly with regard to age, male/female ratio, frequencies of secondary AML, bone marrow blast counts, levels of circulating leukemia cells, or frequencies of cytogenetic abnormalities (Table 1).
- The mutational profile was available for the majority of our patients (Figure S1); a total of 54 mutations were then analyzed, but only 22 of these mutations could be detected for at least one of the patients. The molecular genetic analyses showed no statistically significant differences between the two patient subsets with regard to any individual mutation or the total number of additional mutations (Figure S1).
- The M4/M5 ratio did not differ significantly between the two groups.
- All 25 patients achieved first complete hematological remission after initial induction chemotherapy. Eleven patients had a later relapse (five *NPM1-Ins* patients and six patients without *NPM1*-*Ins*); relapse was then diagnosed ≤26 months after achieving first complete hematological remission for all of them. Three patients achieved a second complete hematological remission; these three could then receive allogeneic stem cell transplantation (one patient with and two patients without *NPM1-Ins*) and became long-term survivors. The overall relapse frequency, time to relapse, and number of patients dying from chemoresistant relapse did not differ significantly between the two patient groups.

Thus, no major differences were detected between the patients with and without *NPM1-Ins* with regard to these clinical and biological characteristics.

### 2.2. Primary AML Cells with NPM1 Insertions Show Increased Levels of Organellar Proteins Involved in Intracellular Trafficking and Transport

**Figure 1:**
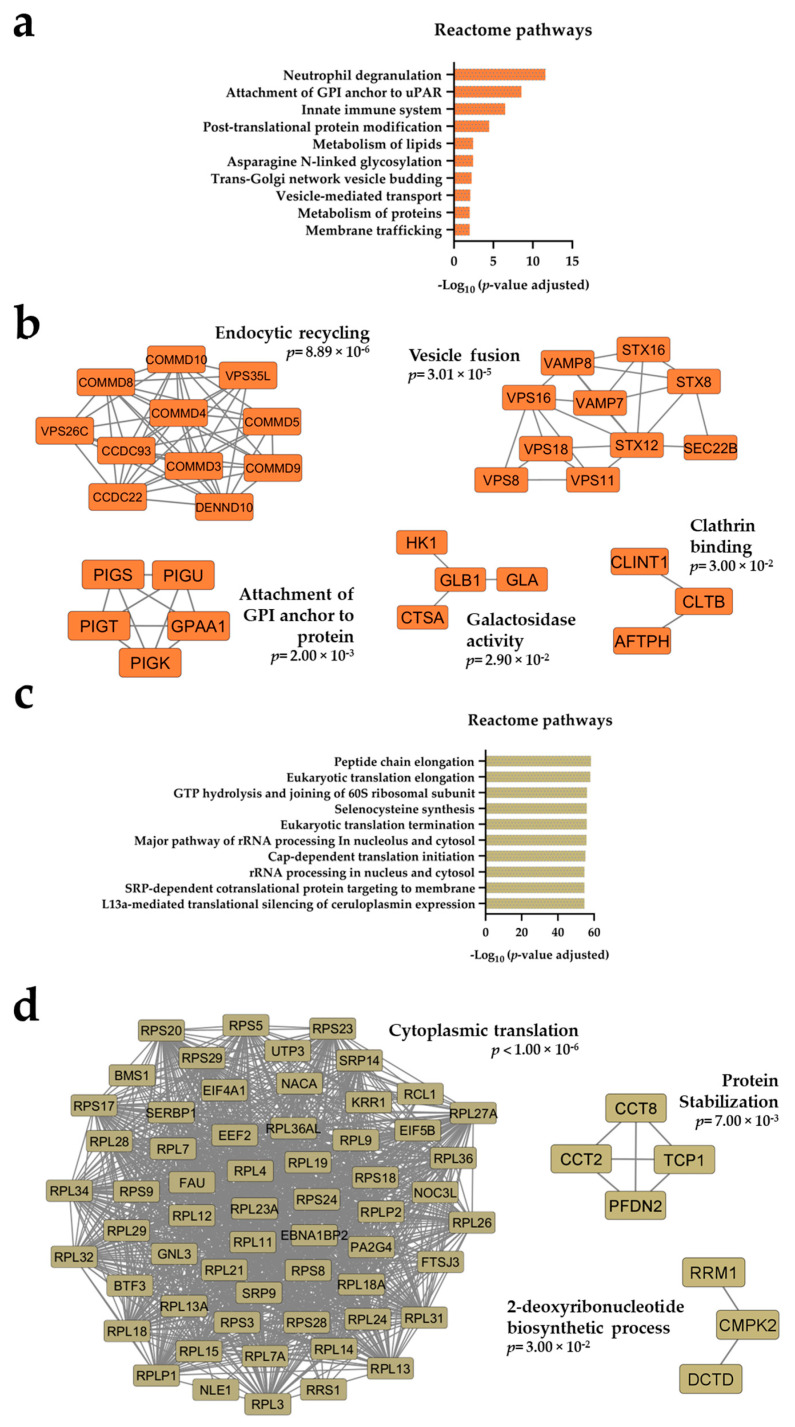
Comparison of the AML cell proteome of FAB-M4/M5 patients with and without *NPM1* insertions (-*Ins*). (**a**,**b**) show significantly enriched Reactome pathways and protein–protein interaction (PPI) networks of differentially expressed proteins with significantly higher expression in *NPM1-Ins* patients; (**c**,**d**) show significantly enriched Reactome pathways and PPI clusters of regulated proteins with a higher expression for patients without *NPM1-Ins* than for *NPM1-Ins* FAB-M4/M5 patients. Significance of PPI cohesiveness is shown with *p*-values of a one-sided Mann–Whitney U test.

We compared the proteomic profiles for primary AML cells derived at the time of first diagnosis with and without *NPM1*-*Ins*. A total of 6781 proteins were identified, and 217 proteins were significantly increased in patients with *NPM1*-*Ins* (Table S2 presents the complete list of increased proteins, Supplementary File S1). The significantly enriched Reactome pathways (Figure 1a) and protein–protein interaction (PPI) networks (Figure 1b) formed by the significantly increased proteins in *NPM1-Ins* patients reflect differences with regard to intracellular endosome/vesicle trafficking and organellar communication. 

The 33 proteins showing significantly increased levels in *NPM1*-*Ins* AML cells and included in the PPI networks (Figure 1b) are important for several key regulatory mechanisms of the cellular functions described above, i.e., endocytosis, communication between intracellular compartments, vesicular transport, or exosome release, as discussed below [26,27,28,29,30,31]. The following is based on the information in Table S3 (this table summarizes important biological/molecular characteristics of each individual network protein) [32,33,34,35,36,37,38,39,40,41,42,43,44,45,46,47,48,49,50,51,52,53,54,55,56,57,58,59,60,61,62,63,64]:

- *Endosomal recycling; the CCC, Retriever, and Commander complexes.* We identified a network that included 11 proteins and is referred to as the endocytic recycling network. This network included several members of the COMMD/CDC22/CDC93 (CCC) complex, including six COMMD molecules (COMMD3/4/5/8/9/10) together with CCDC22, CCDC93, and the Rab GTPase activating DENND10 [32,33,34] (Table 2). This network is important for the recycling of several plasma membrane proteins [34,35,36]. The two last members of this protein interacting network are VPD26C and VPS35L, which form the Retriever complex together with VPS29 [35]. This complex is important for endosomal recycling of several client proteins, including integrins as well as various cell surface receptors [35]. There seems to be an interaction between the CCC and Retriever complexes; they may form a larger assembly and are therefore referred to together as the Commander complex [37].
- *Interactions with the SNARE complex.* The endocytic and secretory pathways consist of various intracellular compartments/organelles [38,39,40] and, as described above, transport of cargo between the various compartments and to/from the cell surface is mediated by membrane-bound vesicles. SNARE (soluble N-ethylmaleimide-sensitive factor attachment protein receptor) proteins mediate membrane fusion of vesicles with their target compartments [39,40]. The SNARE proteins contain characteristic molecular motifs with an approximate length of 60 residues, and they mediate their function through interactions with Sec1/Munc18 (SM) proteins [39]. The SNARE complex is also important for exocytosis/exosome release [41,42], and the complex thereby also becomes important for communication between cells. We observed that AML cells with *NPM1*-*Ins* showed increased levels of six proteins (SEC22B, STX8, STX12, STX16, VAMP7, VAMP8) that interact with SNARE complexes (Table 2 and Table S3).
- *CORVET and HOPS complexes.* The six SEC22b/STX/VAMP proteins listed above formed an interacting network with the four proteins VPS8, VPS11, VPS16, and VPS18 (Table 2 and Table S3). All of these proteins interact with CORVET (class C core vacuole/endosome tethering) and HOPS (homotypic fusion and vacuole/endosome tethering) molecular complexes. These complexes have multiple biological functions in endocytosis, including cooperation with SNARE complexes during membrane fusion, interactions with various GTPases, and attachment of endosomes to the cytoskeleton [43,44]. The complexes have a common class C core subunit that includes VPS11/16/18 [43], i.e., three of the proteins that were increased in *NPM1*-*Ins* AML. The last increased CORVET/HOPS protein was VPS8, which is a specific subunit for CORVET [43]. NPM1 seems to regulate HOPS half-life, and HOPS seem to be a nucleolar bridge between NPM1 and the p19(Arf) tumor suppressor [45]. The HOPS complex is also regarded as an indirect regulator of exosome release [46].
- *CORVET, HOPS, and autophagy inhibition.* There is a close association between the endosome system and autophagy [30]. CORVET and HOPS are important regulators of initial vesicle formation through membrane invagination as well as later endolysosomal trafficking, including regulation of phagocytosis [47,48,49]. We therefore reanalyzed our results from a previous study and compared the antiproliferative effect of the autophagy inhibitor chloroquine for FAB-M4/M5 patients with (20 patients) and without (27 patients) *NPM1-Ins* [50]. However, the antiproliferative effect of chloroquine did not differ between these two FAB-M4/M5 subsets when testing chloroquine 2.5 μM (% ^3^H-thymidine incorporation with chloroquine compared with drug-free controls; median 50.1% versus 47.4% for *NPM1-Ins* and *NPM-wt*, respectively) and chloroquine 5 μM (median 19.9% versus 23.1%).
- *Attachment of GPI (glycosylphosphatidylinositol) anchor to protein.* This network included the five proteins GPAA1, PIGK, PIGS, PIGT, and PIGU (Table 2). These five proteins form the glycosylphosphatidylinositol (GPI) transamidase complex that mediates binding of GPI to target proteins [51,52]. GPI molecules are complex glycophospholipids that serve as membrane anchors for a wide range of proteins [51,52]. The GPI-transamidase is a membrane-bound multi-subunit protein complex of the endoplasmic reticulum where the attachment of GPI to target proteins occurs, but the additional lipid binding occurs upon transport to the Golgi [51,52]. However, GPI-anchored proteins are almost exclusively localized to the cell surface, where they are associated to the plasma membrane through the lipid portion of the anchor [52]. The GPI protein complex is thus involved in preparation of membrane-bound protein forms at intracellular organelles before their transport to the plasma membrane. Increased expression/activity of this complex seems to be involved in carcinogenesis for several malignancies [52].
- *Galactosidase activity.* A small network included four proteins involved in galactosidase activity, i.e., they contribute to the enzymatic profile of lysosomes (Table S3).
- *Clathrin.* Another small network included the clathrin interactor 1 (CLINT1), clathrin light chain beta (CLTB), and aftiphilin (AFTPH) (Table 2 and Table S3) [37,38,51,52,53,54,55,56,57,58,59,60,61,62,63,64]. Clathrin-mediated endocytosis is important for cellular nutrition as well as receptor-mediated endocytosis, membrane recycling, and control of signal pathways [26,30]. Clathrin-coated intracellular vesicles fuse to form early endosomes; these vesicles can later become recycling endosomes that are transported back to the plasma membrane, or they become more mature endosomes that are sorted for (i) fusion with lysosomes, (ii) communication with the Golgi network, or (iii) movement along the microtubule network to the perinuclear area [26,27,30]. Clathrin is important, then, for endosomal functions/organellar transport (e.g., vesicle recirculation) through interactions with adaptor proteins [53,54,55,56], including CLINT1 and AFTPH, which are important for the AP-1 adaptor complex and its interaction between endosomes and the Golgi apparatus [57]. Aftiphilin, in addition, interacts with the AP-2 complex, which is important in clathrin-mediated endocytosis [57]. Finally, animal studies suggest that clathrin can be important in AML leukemogenesis [58,59]. Thus, this small protein interaction network should be regarded as important for the regulation of clathrin-dependent endocytosis.

Taken together, these differences in the proteomic profiles strongly suggest that the intracellular endosomal transport and recycling of cell surface molecules differs between AML cells without and with *NPM1-Ins*.

### 2.3. Patients without NPM1-Ins Show Increased Levels of Proteins Involved in Regulation of Transcription/Translation, Especially Ribosomal Proteins, Transcriptional Regulators, and Chaperones

A total of 132 proteins were significantly increased for primary AML cells derived from patients without *NPM1*-*Ins* (Supplementary File S1). A complete list of these proteins is presented in Table S4. Both the Reactome pathway (Figure 1c) and PPI analyses (Figure 1d) identified several proteins being important in transcription/translation and showing increased levels in patients without *NPM1*-*Ins* (Table 3). Based on the discussion below [65,66,67,68,69] and the additional information in Table S5 [65,66,67,68,69,70,71,72,73,74,75,76], these network proteins can be classified as follows:

- Several ribosomal proteins differed significantly between the two groups, especially 60S proteins (29 proteins) but also 40S proteins (15 proteins).
- Several of the proteins are involved in RNA binding/processing (eleven proteins), transcription (four proteins), and protein synthesis (translation/elongation, seven proteins). Five of the ribosomal/transcription/translation proteins are located in the nucleus and four of these in the nucleolus.
- Four proteins are chaperons, i.e., they are involved in protein quality control.
- Three proteins are involved in nucleotide metabolism (CMPK2, DCTD, RRMI).
- A heterogeneous group consisting of nine other proteins was identified; it included three proteins involved in regulation of cytoskeleton/autophagy/apoptosis (CCT2, CCT8, TCP1) and one involved in epigenetic regulation.
- Only two of these network proteins have previously been identified as possibly being involved in leukemogenesis (GNL3, PA2G4) [65,66,67], and two additional proteins are possibly involved in carcinogenesis (RPL7A, RPL34) [68,69].

Thus, primary AML cells with and without *NPM1*-*Ins*, being derived at the time of first diagnosis, differ with regard to regulation of RNA transcription/ribosomal functions/protein synthesis. 

### 2.4. Comparison of Phosphoproteomic Profiles for FAB-M4/M5 Cells with and without NPM1-Ins; the Phosphoproteomic Differences Are Less Extensive but Extend the Proteomic Differences

**Figure 2:**
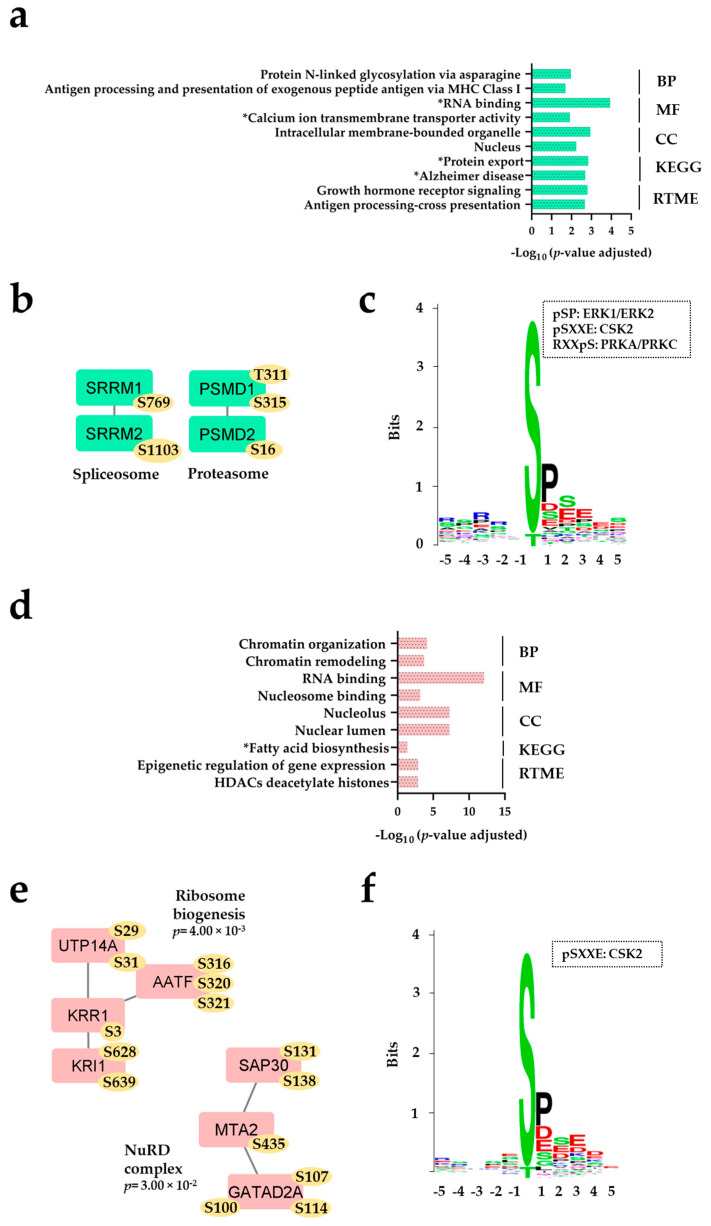
The AML cell phosphoproteome of FAB-M4/M5 patients. All analyses are based on those phosphosites that differed significantly between the two groups; the upper part (**a**–**c**) presents the results for protein sites with increased phosphorylation levels for patients with *NPM1*-*Ins*, and the lower part (**d**–**f**) presents the results for patients without *NPM1*-*Ins*. For both groups we show Reactome pathway analysis (**a**,**d**), PPI analysis (**b**,**e**), and sequence motif analysis of the ±5 amino acids flanking the differentially regulated phosphorylation sites (**c**,**f**). * Significance was only detected with unadjusted *p*-values < 0.05.

We did a phosphoproteomic comparison of FAB-M4/M5 AML cells with and without *NPM1-Ins*. A total of 12,309 phosphosites on 3003 proteins were detected, and relatively few phosphosites differed significantly between the two groups (Figure 2, Supplementary File S2). A complete list of phosphosites that were significantly increased in patients with and without *NPM1-Ins* is given in Tables S6 and S8, respectively, and more detailed descriptions of biological function for those proteins/phosphosites identified in the corresponding PPI networks are given in Table S7. Patients with *NPM1-Ins* showed increased phosphorylation, especially for proteins involved in organellar communication/transport and RNA binding, whereas patients without *NPM1-Ins* showed increased phosphorylation, especially for proteins involved in transcriptional/epigenetic regulation (Figure 2a,d). Only four small PPI networks of differentially regulated phosphosites were identified, including 19 phosphosites of 11 proteins (Figure 2b,d).

- *Patients with NPM1-Ins (70 sites with increased phosphorylation).* Several of the identified Reactome pathways reflect increased phosphorylation of proteins involved in membrane functions/intracellular transport, e.g., antigen processing/presentation, calcium ion transmembrane transporter activity, and intracellular membrane-bound organelle and protein export (Figure 2a). However, only two interacting protein pairs could be identified by PPI analyses. These pairs reflect different functions of two spliceosomal and two proteasomal proteins, respectively (Figure 2b, Tables S6 and S7). Both proteasomal proteins are parts of the 19S molecular complex. A previous study suggests that proteins within this regulatory 19S subunit (including non-ATPase subunits) can be involved in solid tumor carcinogenesis and even in leukemogenesis in human AML [77].
- *Patients without NPM1-Ins (76 sites with increased phosphorylation).* Most of the identified phosphosites reflect differences between proteins involved in epigenetic/transcriptional regulation, e.g., the five Reactome pathways of chromatin organization/remodeling, RNA/nucleosome binding, nucleolus, epigenetic regulation of gene expression, and histone deacetylase (HDACs) deacetylate histones (Figure 2d). The PPI analysis identified two small protein networks. One network included four proteins (eight phosphosites) referred to as ribosome biogenesis; two of these proteins seem to have additional indirect effects on regulation of apoptosis through effects on intracellular signaling (Figure 2e) [78]. Two of these three other proteins are linked to AATF within this network, the AATF protein being involved in ribosome biogenesis, checkpoint control/DNA repair, and regulation of apoptosis [79,80,81,82], and even being regarded as a possible therapeutic target in cancer treatment [83]. The second network included three proteins and is referred to as the NuRD complex. Two of these proteins function as deacetylases (SAP30, MTA2), and the proteins in this network modulate the effects of both p53 (MTA2) and STAT3 (GATAD2A) (Tables S7 and S8) [84,85]. The NuRD complex is a chromatin remodeling complex [86,87]. Lysine-specific demethylase 1A (LSD1) is a constituent of critical transcription repressor complexes, including the nucleosome remodeling and deacetylase (NuRD) complex. Experimental studies suggest that LSD1 targeting is a possible therapeutic strategy in AML, leading to impaired self-renewal and proliferation as well as increased differentiation and apoptosis of primary AML stem cells, especially AMLs with KTM2A and RUNX1 rearrangements or erythroid/megakaryoblastic differentiation block [88].
- *Sequence motif analyses*. Casein kinase 2 (CSK2) seems particularly important for the sites showing a high degree of phosphorylation in cells without *NPM1-Ins* (Figure 2f), whereas several kinases seem important in *NPM1*-*Ins* patients (Figure 2c; Erk1/2, CSK2, protein kinase A/C). Kinase enrichment analysis with KEA2 confirmed the increased activity of serine/threonine-protein kinases, MAPK14 (*p* = 0.03) and MAPKAPK1B (*p* = 0.04), in patients with and without *NPM1-Ins*, respectively.

Taken together, these observations further support our previous conclusion that FAB-M4/M5 patients with and without *NPM1*-*Ins* differ with regard to endosomal functions/intracellular transport and transcriptional regulation. Several of these proteins may be involved in AML leukemogenesis and/or chemoresistance (see above).

### 2.5. The Heterogeneity of FAB-M4/M5 Patients with and without NPM1-Ins

To further investigate the heterogeneity of FAB-M4/M5 AML cells, we did hierarchical clustering analyses based on the differentially expressed proteins that, in addition, were identified by the PPI network analyses (Figure 1). 

**Figure 3:**
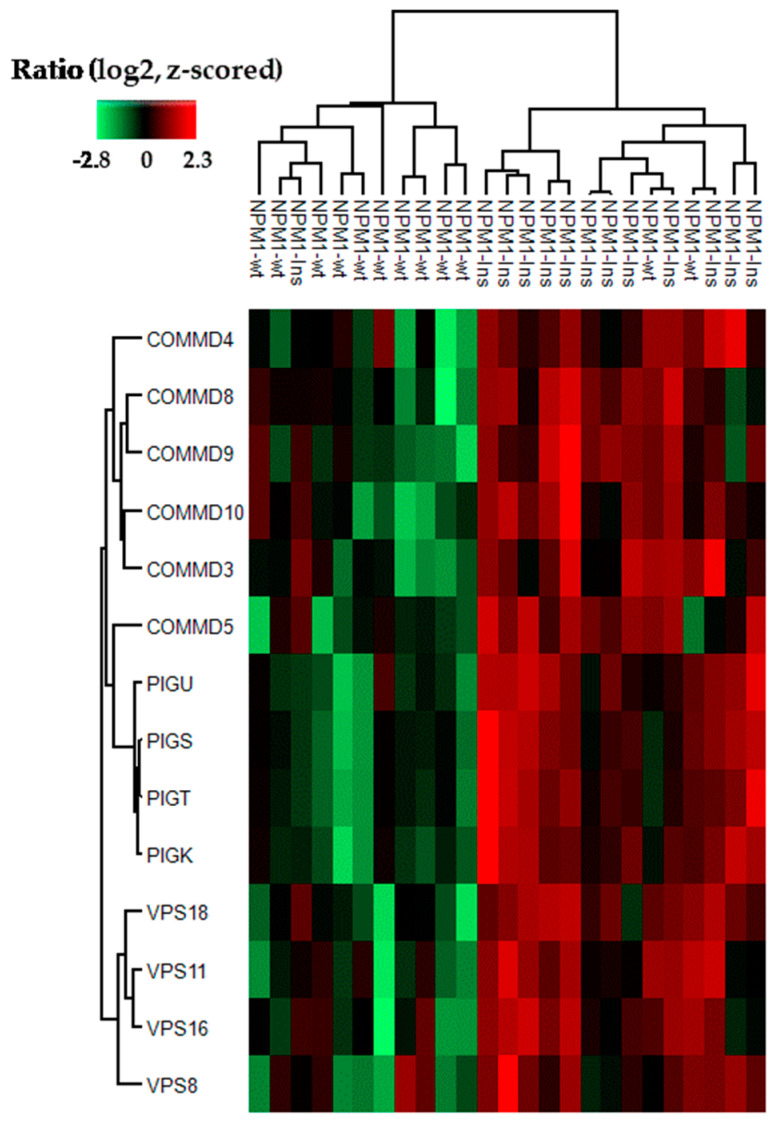
Unsupervised hierarchical clustering of FAB-M4/M5 patients with and without (referred to as *NPM1-wt* in the figure) *NPM1-Ins*. The analysis is based on the expression of COMM domain-containing proteins, GPI transamidase proteins, and vacuolar sorting proteins showing differential expression in ANOVA and, in addition, being included in PPI networks.

The clustering analysis based on network proteins showing increased levels in the *NPM1-Ins* patients showed that all except one of the patients with *NPM1-Ins* clustered together in the same main patient cluster, characterized by generally high levels of these proteins (Figure 3); this difference in distribution of *NPM1-Ins* patients between the two main patient clusters is highly significant (Fisher’s exact test, *p* = 0.0002). The only exceptional *NPM1-Ins* patient was a 47-year-old woman with de novo AML, normal karyotype, and *NPM1-Ins* but no genetic *Flt3* abnormality. Thus, this patient showed no specific clinical or biological differences from the other patients with *NPM1-Ins*. Finally, only two of the patients without *NPM1-Ins* clustered together with the *NPM1-Ins* patients.

We also did additional hierarchical clustering analyses based on the differentially expressed large (Figure S2a) and small (Figure S2b) ribosomal subunit proteins. Although most *NPM1*-*Ins* patients generally clustered close to each other, several exceptional *NPM1-Ins* patients were identified in both analyses.

Taken together, these results suggest that a specific metabolic proteomic pattern is very common in *NPM1-Ins* patients, but it is not specific for these patients and is also identified in FAB-M4/M5 patients without *NPM1-Ins*. The patient subset without *NPM1-Ins* seems to be more heterogeneous, and this is not surprising because these patients probably do not have any common genetic abnormality (Table S1).

### 2.6. The Profiles of Differentially Expressed Proteins When Comparing AML Cells with and without NPM1-Ins; the Profiles of Monocytic AML Cells with and without NPM1-Ins Differ from Each Other but Both Profiles Differ from Normal CD34^+^ Bone Marrow Cells

Using label-free quantification (LFQ), we compared the profiles of differentially expressed proteins for 13 monocytic *NPM1-Ins* AML cell populations, 12 AML cell populations without *NPM1-Ins*, and eight normal CD34^+^ bone marrow stem/progenitor cell populations. We first compared the profiles of the differentially expressed proteins that formed the PPI networks shown in Figure 1b,d and were quantified in all patients as well as in CD34^+^ cells, i.e., the nine proteins involved in intracellular vesicular trafficking, the 17 large ribosomal subunit proteins, and the eight small ribosomal subunit proteins. We performed a principal component and unsupervised hierarchical clustering analysis (Figure S3a,b). All eight normal CD34^+^ cells clustered together and distinctly from AML cell populations. These observations illustrated that AML-associated profiles of differentially expressed PPI networks differ from the expression profile of the normal CD34^+^ cells, and this seems to be true both for cells with and without *NPM1-Ins* mutations. Furthermore, we compared the profiles of 426 differentially expressed label-free proteins for *NPM-Ins* patients, patients without *NPM1* mutations, and normal CD34^+^ bone marrow cells. Both principal component and unsupervised hierarchical clustering (Figure S4a,b) showed that normal CD34^+^ bone marrow cells showed striking similarities in their expression profiles and localized together and separated from the AML cells in both analyses. 

Taken together, these analyses illustrate that the profiles of differentially expressed proteins in monocytic AML cells both with and without *NPM1-Ins* differ from the corresponding profiles in normal CD34^+^ bone marrow cells, and this was observed both when comparing all differentially expressed proteins and when only the proteins identified in PPI networks were included in the clustering analyses. 

## 3. Discussion

In the present study, we reanalyzed our previously published LC-MS/MS-based proteomic and phosphoproteomic cohort of primary pretreatment AML cells derived at the first time of diagnosis [25]. We selected the subset of patients classified as FAB-M4/M5 for our present study, as pointed out in Section 2.1. This study was based on a careful standardization of both the methods for the handling of cell samples and the proteomic/phosphoproteomic analyses, and our bioinformatical analyses have a focus on protein networks/biological functions.

Our study is based on examination of relatively young and fit AML patients that could receive conventional intensive and potentially curative chemotherapy, and they represent a consecutive group from a defined geographical area [25]. Enriched leukemic cells were derived at the first time of diagnosis, and all patients received initial conventional intensive and potentially curative chemotherapy; only patients with later relapse were treated with allogeneic stem cell transplantation if they reached a second complete hematological remission. For the present study, we selected those 25 patients classified as FAB-M4/M5, among the consecutive patients, and the FAB-M4/M5 patients with and without *NPM1*-*Ins* did not differ significantly with regard to clinical and biological characteristics at the time of first diagnosis. Approximately half of the patients in each group had a later relapse (Table 1 and Table S1).

All patients with and without *NPM1-Ins* included in this study had at least 20% AML blasts in their bone marrow and thereby fulfilled the WHO 2016 criteria for the diagnosis of AML [89]. According to the WHO 2022 criteria, the diagnosis of *NPM1-Ins* AML can be made with lower blast counts; this is due to the previous observation that *NPM1-Ins* cases with lower blast percentage, classified as *NPM1*-mutated MDS, rapidly progress to AML with >20% blast counts [1,90,91]. However, the *NPM1* mutations show a high stability even when comparing AML cells at first diagnosis and at the time of relapse [89,92]. Thus, even though the WHO criteria for the diagnosis of *NPM1-Ins* AML have changed since our original study of the present patient cohort, we regard our present observations still to be representative for *NPM1-Ins* AML. 

*NPM1-Ins* is associated with favorable prognosis [1,2,5,6]. Even though the frequency of AML relapse in first remission was slightly lower for patients with versus patients without *NPM1-Ins* (five out 13 versus six out of 12, respectively), this difference did not reach statistical significance. The time to relapse and number of patients dying from chemoresistant relapse did not differ significantly between the two patient groups either. The explanation for this lack of statistically significant differences between our patient subsets with regard to relapse/survival is probably that the two groups are too small to allow reliable analyses of survival/relapse risk.

AML cells with monocytic differentiation constitute a heterogeneous group with regard to genetic abnormalities, and thereby also prognosis, after intensive conventional antileukemic therapy [7,8,9,10,11,12]. Such a heterogeneity was also seen for our present patients, but as expected our patients included a large subset with *NPM1*-*Ins*. However, despite the genetic heterogeneity the monocytic morphology (i.e., FAB-M4/M5) is generally associated with resistance to Bcl-2 inhibitors (i.e., venetoclax) and possibly also CDK4/6 inhibitors, whereas these cells are susceptible to BET inhibitors [22,23,24]. Monocytic differentiation is also associated with a different mitochondrial energy metabolic profile that is important for the development of the venetoclax resistance [93]. Primary AML cells show the following metabolic characteristics:

- AML cells, in general, have an increased mitochondrial mass but also a mitochondrial dysfunction, with reduced maximal respiratory capacity [94], signs of oxidative stress, and increased levels of reactive oxygen species (ROS) [94,95].
- Bcl-2 inhibitors (i.e., venetoclax) inhibits oxidative phosphorylation and energy production in primary AML cells [93,96], and in vivo studies have shown that such treatment inhibits the electron transport chain, increases cellular ROS, and decreases glutathione levels. Thus, metabolic effects involving oxidative phosphorylation seem to be important for the antileukemic effects of venetoclax in cells that depend on Bcl-2 for survival [93,97].
- However, patients with the FAB-M4/M5 variants of AML have higher basal ROS levels [98]; this cannot be caused by mutation in members of electron transport chain complexes [99] but is rather caused by upregulation of genes important for oxidative phosphorylation [22].
- Our present results show only minor differences in mitochondrial energy metabolism between the two FAB-M4/M5 subsets, with hexokinase being the only protein involved in energy metabolism.

Even though we compared two different genetic subsets of FAB-M4/M5 AML (i.e., with and without *NPM1-Ins*), these two subsets did not show any major proteomic or phosphoproteomic differences with regard to energy metabolism (only hexokinase levels being different, see Figure 1, Table 2 and Table S3). This is consistent with the hypothesis that FAB-M4/M5 AML cells with and without *NPM1-Ins* show extensive similarities in their regulation of mitochondrial energy metabolism.

The bromodomain and extraterminal domain (BET) protein family includes four epigenetic regulators (BET2-4, BRDT), and BET4 has been identified as a growth-enhancing mediator in AML [23,100]. BET-inhibitor-resistant AML cells show decreased markers of differentiation, whereas monocytic differentiation is associated with sensitivity to BET inhibitors [23]. Furthermore, monocytic differentiation is also associated with resistance to CDK4/6 inhibitors [23]. Finally, a previous study described an association between monocytic differentiation markers and high sensitivity to bortezomib-mediated apoptosis [93], but neither this nor another study could detect any significant association between FAB-M4/M5 and the antileukemic effect of proteasome inhibitors [101,102]. Another study of AML cell lines found that bortezomib sensitized myelomonocytic cells to TRAIL-induced apoptosis [103]. Taken together, these observations further emphasize the importance of AML cell differentiation for sensitivity to various targeted therapies and not only Bcl-2 inhibition. 

Endosomes are plasma-membrane-derived cellular organelles that function as temporary transport vesicles [26,27,28,29,30,31]. Extracellular and plasma membrane macromolecules are then internalized by primary endocytic vesicles formed by invagination of the cellular membrane; these vesicles fuse with each other to form the larger early endosomes that are localized mainly at the periphery of the cytoplasm, where they can move along the microtubule network and recirculate parts of their cargo back to the plasma membrane [31]. Alternatively, early endosomes can communicate with the trans-Golgi network via bidirectional molecular exchange, or they can mature to late endosomes and move to the perinuclear area. The late endosomes may also (i) acquire intraluminal vesicles through interactions with various organelles, (ii) function as secondary sorting stations of cargo that is delivered to various destinations, or (iii) fuse with lysosomes [31]. Late endosomes/multivesicular bodies can also fuse with the plasma membrane and thereby release their molecules and their intraluminal vesicles as exosomes [29].

A main characteristic of FAB-M4/M5 patients with *NPM1*-*Ins* was altered regulation of endosomal functions/intracellular transport (Figure 1 and Figure 3). As discussed above, the *NPM1-Ins* cells showed increased levels of several proteins that are a part of or interact with molecular complexes regarded to be key regulators in intracellular trafficking. Furthermore, our clustering analysis showed that the majority of *NPM1-Ins* cells had proteomic profiles with generally high levels of all these proteins. In contrast, FAB-M4/M5 patients without *NPM1*-*Ins* show relatively high levels of several proteins important for transcription/translation/chaperoning, including several ribosomal proteins. Thus, even though monocytic AML cell differentiation is associated with differences in responsiveness to certain targeted therapies (e.g., Bcl-2, BET, CDK4/6, and possibly proteasomal inhibitors), and this seems to at least partly be due to common metabolic characteristics [22,93], our present study demonstrates that FAB-M4/M5 patients also show a proteomic heterogeneity with regard to fundamental cellular functions. 

Targeting of cellular metabolism is now regarded as a possible strategy in AML [104,105], and our present results are thus consistent with the hypothesis that this strategy can be used to target metabolic mechanisms that are common for FAB-M4/M5 AML. On the other hand, targeting of cellular membrane molecules/intracellular transport/organellar functions may be more effective for patients with *NPM1*-*Ins*, e.g., V-ATPase inhibition [106] or inhibition of key regulatory complexes of endocytosis/vesicular trafficking functions that show increased expression in FAB-M4/M5 *NPM1-Ins* patients, as described in Section 2.2 (see also several recent reviews [26,27,28,29,30,106]). Briefly, these molecular regulators are involved in several cellular processes:

- The endosomal system is involved in the regulation of intracellular signaling (e.g., Notch signaling) [30,107].
- Intracellular transport is important for the communication between neighboring cells through the release of exosomes [29].
- Endocytosed vesicles form early endosomes that can recirculate their content (e.g., cell surface receptors) back to the plasma membrane/cell surface [26,30].
- Alternatively, early endosomes can further develop into late endosomes [29,30]; this development is associated with increased intravesicular acidification, moving to the perinuclear area, and interactions with other intracellular organelles like the endoplasmic reticulum and the trans-Golgi apparatus [27,30].
- Late endosomes may also take up vesicles and form multivesicular bodies/late endosomes [29], and they may fuse with lysosomes or become exosomes [28,29].
- Alternatively, endosomes can fuse with lysosomes, and modulation of the lysosome system may contribute to malignant cellular transformation and/or the further development to malignant disease [30].
- There is a close association between the endosome system and autophagy, and thereby also between these and cellular metabolism [30]. This is also reflected in our present results; several of the differentially expressed proteins are also involved in autophagy, but despite this we could not detect any significant difference in the antiproliferative capacity of the autophagy inhibitor chloroquine when comparing monocytic AML cells with and without *NPM1-Ins*.

Thus, the increased levels of endosome-associated proteins in *NPM1*-*Ins* AML cells will probably contribute to the unique biological characteristics of these cells, and these mechanisms may also represent possible therapeutic targets. First, targeting of V-ATPase will modulate the regulation/acidification of vesicular pH, but it is also a regulator of other endosomal/lysosomal functions, e.g., membrane fusion and budding, vacuole fission/fragmentation, and lysosomal exocytosis with protease release [108]. ATPase targeting will also modulate receptor recycling and influence important intracellular signaling pathways, e.g., mTOR, Notch, and Wnt/β-cathenin signaling [106,108,109,110]. Second, an alternative strategy may be to target important consequences of the altered endosomal/exosomal/lysosomal regulation, e.g., inhibition of intracellular signaling or autophagy. Finally, *NPM1*-*Ins*, high mRNA [111], protein V-ATPase expression [25], monocytic differentiation, and high extracellular cytokine release [111] are all associated with favorable prognosis in human AML. Taken together, these observations suggest that targeting of intercellular communication through inhibition of V-ATPase/extracellular secretion should be considered for *NPM1-Ins* FAB-M4/M5 AML.

Endocytosis is important for the initial internalization of pharmacological nanoparticles to malignant cells, and thus it will be essential to avoid later endosomal degradation [112,113]. Differences between AML patients with regard to regulation of endocytosis and vesicular trafficking in the leukemic cells may therefore require individualization of nanoparticle-based therapeutic strategies in AML.

We analyzed the proteomic profiles of FAB-M4/M5 patients based on differentially expressed proteins involved in endocytosis/transport/intracellular trafficking (Figure 3) and ribosomal proteins (Figure S2). *NPM1-Ins* patients showed an endosomal/transport profile that was common for the large majority of mutated cases and uncommon for patients without *NPM1-Ins*. Ribosomal protein analysis also identified several patient subsets that differed in their ribosomal protein profiles, but several *NPM1-Ins* patients then showed similar profiles to patients without *NPM1-Ins*. Thus, endosomal/trafficking/transport proteins seem to show a more striking difference between the patients with and without *NPM1-Ins* than the ribosomal protein profile.

We compared the proteomic profiles for monocytic AML cells with and without *NPM1-Ins* to normal CD34^+^ bone marrow cells. These comparisons were based on the differentially expressed proteins identified by the comparison of the two AML cell/patient subsets. As expected, these alternative bioinformatical comparisons further illustrate that the two AML cell subsets differ in their profiles of differentially expressed proteins. However, the AML-associated profiles based on all differentially expressed proteins and based on the interacting protein networks seem to differ from the corresponding profiles of normal CD34^+^ stem/progenitor AML cells.

A major limitation of our present study is that we only investigated AML cells showing monocytic differentiation (i.e., FAB-M4/M5). It will be important to clarify whether the observed proteomic/phosphoproteomic characteristics of our *NPM1-Ins* AML cells are seen only in combination with monocytic differentiation, or whether it is a general characteristic of *NPM1*-*Ins* AML cells. This will be important to know with regard to the possible use of new targeted therapies in defined subsets of AML. Furthermore, we did not confirm our observations with alternative analytical strategies but, in our opinion, a highly standardized and documented proteomic/phosphoproteomic methodology is reliable (see also Section 4.1 and Discussion section). We would also emphasize that we focus on protein interactions and network analyses/biological processes that cannot be explained by coincidence alone, rather than single proteins, and closely related processes/network functions are identified by using different bioinformatical approaches. A final limitation is that our present study did not include new functional data suggesting that the identified proteomic differences in vesicular trafficking, metabolism, and/or transcriptional regulation are functionally important for FAB-M4/M5 cells with and without *NPM1-Ins*. However, previous studies have demonstrated that, for example, the CORVET and HOPS complexes seem to have a general function in different functions requiring intracellular trafficking (e.g., early vesicle formation, phagocytosis, endo-lysosomal trafficking, autophagy), and the observation that such processes are altered by NPM1 interacting agents, especially in *NPM1-Ins* AML cells, strongly suggests that our present observations are functionally relevant [114]. Monocytic *NPM1-Ins* AML cells are characterized by a specific gene expression profile [115], and NPM1 is also important in ribosomal biogenesis [116]. Thus, our present results represent a detailed characterization of the molecular mechanisms behind/associated with fundamental characteristics (regulation of transcription/translation and trafficking) previously described of *NPM1-Ins* cells. The lack of association between effects of autophagic inhibition and *NPM1* status in monocytic AML cells is not surprising because autophagy does not seem to have a (strong) regulatory effect on initial steps of vesicle formation/invagination [48].

Analyses of proteomic and/or phosphoproteomic profiles may become useful for the evaluation of the antileukemic effect of AML treatment. A recent study described that in vivo reduction of the ERK1/2-p38 signaling early after the start of conventional induction therapy was associated with long-term survival/relapse risk [117]. Proteomic or phosphoproteomic evaluation may then represent a strategy for a broader early evaluation of molecular treatment responses and can then be used to identify alternative single marker or biomarker profiles that can be used for early evaluation of treatment responses. An alternative strategy could be to identify a panel of relevant biomarkers and to make an individualized selection of the optimal biomarker(s), based on the molecular biology of the patients AML cells. Furthermore, monocytic differentiation induction seems to be a part of the antileukemic effect of various new targeted therapies, including agonists to the aryl hydrocarbon receptor [118] as well as inhibitors of IDH [119,120,121], Flt3 [122,123], histone deacetylases [124], LSD1L [125,126], and exportin 1 [127]. This differentiation induction has usually been evaluated by the expression of a single or few monocyte markers, and for some studies also by morphology. Our present study demonstrates that monocytic differentiation can be associated with different proteomic/phosphoproteomic profiles. Future studies, therefore, have to characterize in more detail the therapy-induced monocytic differentiation to clarify whether the monocytic phenotype differs between various targeted therapies; this will be important for characterization of these treatment responses and for the future design of optimal combinations of targeted therapies. 

## 4. Materials and Methods

### 4.1. Patients and Sample Collection

We re-analyzed our previously published LC-MS/MS-based proteomic and phosphoproteomic cohort of primary AML cells, derived at the time of diagnosis from 41 Caucasian patients [25]. These 41 patients represent a consecutive group of relatively young patients that were fit for intensive and potentially curative antileukemic treatment. Patients with the acute promyelocytic leukemia (APL) variant were excluded from this previous study. The study should be regarded as population-based because our department was responsible for diagnosis and treatment of AML in a defined geographical area during this defined time period. The clinical and biological characteristics of the FAB-M4/M5 patients included in the present study are presented in Table 1 and Table S1. All of the patients had at least 20% AML blasts in the bone marrow at the time of sampling, and all patients classified as relapse-free were observed for at least seven years. 

Enriched primary AML cells were density gradient separated from the peripheral blood (PB) of untreated patients with blast counts (leukemic cells) exceeding 80% of the circulating leukocytes. All samples were cryopreserved according to the same highly standardized protocol [128] and samples were stored in liquid nitrogen for the whole storage period. The duration of the storage period did not differ significantly for cells with and without *NPM1-Ins*, and the procedures for thawing and lysate preparation were highly standardized as described previously [25,129,130,131,132,133,134,135]. Furthermore, in our previous study, the general quality of our proteomic data was confirmed by additional Western blot analyses of two differentially expressed proteins (CDK2, ERK1/2) and three differentially expressed phosphosites (CDK2T160, ERK1/2T202, ERK1/2 Y204).

Normal CD34^+^ bone marrow stem/progenitor cells were derived from eight individuals without hematological disease (PromoCell GMBH; Heidelberg, Germany); these cells were derived from four men and four women, and their age did not differ significantly from the AML patients of this study. All control individuals were Caucasians. Quantitative proteomics of the AML patients and CD34^+^ cells was performed according to the LFQ approach, as described in our earlier study [25].

Protein quantification was performed by combining protein lysates from a heavy-marked AML-super SILAC (Stable Isotope Labeling by Amino acids in Cell culture) mixture [135] with the same amount of lysate from each patient sample before trypsin digestion and subsequent LC-MS/MS analysis. Detailed methods description [131] and patient information on FAB type, cytogenetic, and mutational analysis from the time of diagnosis is given in our previously published cohort of 41 AML patients [25]. All raw data and MaxQuant output files can be found in the ProteomeXchange consortium with the dataset identifier PXD014997.

### 4.2. Subclassification of AML Patients Based on Leukemic Cell Differentiation

The FAB classification is regarded as a standardized and well-described system to characterize and classify AML patients with regard to the differentiation status of their leukemic cells [21,22]. In our present study, we defined monocytic differentiation as FAB-M4/M5 classification according to this system. 

### 4.3. Molecular Genetic Analyses

Analyses of *Flt3* and *NPM1* mutations have been described previously [136,137]. Submicroscopic mutational profiling of 54 genes frequently mutated in AML was undertaken by the Illuminas TruSight Myeloid Gene Panel and sequenced using the MiSeq system and reagent kit v3 (all from Illumina, San Diego, CA, USA) [137]. 

### 4.4. Statistical and Bioinformatical Analyses

The Perseus 2.0.7.0 bioinformatics platform was used for functional and statistical analysis of the proteomics data [138]. Patient subgroups were normalized using width adjustment. Proteins and phosphosites (localization probability > 0.75) with a minimum of three individual SILAC ratios for each patient group were selected for statistical analyses. ANOVA multiple sample test was performed with a threshold *p*-value < 0.05 to test for significant difference between means for the proteins and phosphosites between the patient subsets. A post hoc Turkey’s Honest Significance Difference (HSD) test with FDR < 0.05 was performed on the ANOVA-significant pairs of protein and phosphosites. Reactome pathway, Gene Ontology (GO), and KEGG pathway enrichment analyses were obtained with the Enrichr gene set search engine [139,140,141,142]. Protein–protein interaction (PPI) network analyses were performed with STRING database version 11.5 [143]. Phosphosite motif analyses were done with the web-based WebLogo application and with the kinase enrichment analysis tool KEA2 [144,145]. Venn diagrams were generated using BioVenn [146].

Perseus software 2.0.7.0 was also used for unsupervised hierarchical clustering analyses based on significantly differing proteins; the analyses were done using the Euclidean correlation function and complete linkage. The Fisher’s exact test was used for analysis of categorized data. 

## 5. Conclusions

Monocytic differentiation is associated with the responsiveness to certain targeted therapies (i.e., Bcl2, BET, and CDK4/6 inhibition) but, despite such pharmacological similarities, FAB-M4/M5 patients with and without *NPM1*-*Ins* differ with regard to transcriptional/translational regulation and endosomal functions/intracellular transport.

## Figures and Tables

**Table 1:** A summary of clinical and biological characteristics of our FAB-M4/M5 patients with and without *NPM1* insertions (-*Ins*). Categorized data were analyzed by the Fisher’s exact test and continuous data by the Mann–Whitney U test.

Parameter	Patients with *NPM1*-*Ins* (*n* = 13)	Patients without *NPM1*-*Ins* (*n* = 12)	*p*-Value
Male/female	7/6	8/4	0.69
Age (years, median, and range)	48 (29–65)	53 (18–62)	0.81
Secondary AML (number)	5	2	0.10
Bone marrow blast count (%)	83 (50–97)	50 (25–90)	0.19
Peripheral blood blast level (×10^9^/L)	36.9 (8.6–101)	33.2 (3.8–351)	0.76
FAB-M4/FAB-M5 (number)	6/7	8/4	0.42
Normal karyotype (number)	10	5	0.11
Abnormal karyotype (favorable/intermediate/adverse) ^1^	1/2/0	4/1/2	
Flt3-internal tandem duplication (ITD) (number)	4	2	0.64
Relapse after remission induction (fraction)	5/13	6/12	0.43
Time from diagnosis to relapse (months, median and range)	11 (7–18)	11 (6–26)	0.58

^1^ Classified according to the ELN criteria [2].

**Table 2:** Proteins showing significantly increased levels in AML cells with *NPM1-Ins*: a summary of proteins included among the identified protein–protein interaction (PPI) networks presented in Figure 1b.

Network/Complex	Proteins
Commander complex	The CCC complex: COMMD3, COMMD4, COMMD5, COMMD8, COMMD9, COMMD10, CCDC22, CCDC93, DENND10 The Retriever complex: VPS26C, VPS35L
SNARE interactions	SEC22B, STX8, STX12, STX18, VAMP7, VAMP8
CORVET/HOPE complex	VPS8, VPS11, VPS16, VPS18
Transamidase; GPI anchoring	PIGS, PIGU, PIGT, PIGK GPAA1
Galactosidase	HKI, GLB1, GLA, CTSA
Clathrin	CLINT1, CLTB, AFTPH

**Table 3:** Proteins showing significantly increased levels in AML cells without *NPM1*-*Ins*; a summary of proteins included in the identified PPI networks presented in Figure 1d.

Function/Localization	Proteins
Ribosomal 40S complex (structure/function; 15 proteins)	BMS1, EBNA1BP2, FAU RPS 1, RPS3, RPS5, RPS8, RPS9, RPS17, RPS18, RPS20, RPS23, RPS24, RPS28, RPS29
Ribosomal 60S complex (structure/function; 29 proteins)	NLE1 RPL 2, RPL 3, RPL4, RPL7, RPL7A, RPL9, RPL11, RPL12, RPL13, RPL14, RPL15, RPL18, RPL18A, RPL19, RPL21, RPL23A, RPL24, RPL26, RPL27A, RPL28, RPL29, RPL31, RPL32, RPL34, RPL36, RPL36AL RPLP1, RPLP2
Ribosome, others (4 proteins)	EEF2, EIF5B, GNL3, NACA, SERBP1
RNA binding/processing (11 proteins)	BTF3, EBNA1BP2, EEF4A1, FTSJ3, KRR1, NACA, PA2G4, RCL1, SRP14, SRP9, UTP3
Transcription (4 proteins)	BTF3, PA2G4, RPLP1, RPLP2
Protein synthesis (translation/elongation; 7 proteins)	EEF2, EEF4A1, EIF5B, NACA, RPSR, RPLP1, RPLP2
Nucleus (2 proteins)	GNL3, SERBP1
Nucleolus (5 proteins)	EBNA1BP2, FTSJ3, GNL3, NLE1, RCL1
Protein stabilization ( chaperones; 4 proteins)	CCT2, CCT9, TCP1, PFDN2
Nucleotide metabolism (3 proteins)	CMPK2, DCTD, RRM1
Other proteins (9 proteins; biological function/relevance given in parenthesis)	CCL15 (chemokine), NLE1 (Notch signaling), PA2G4 (epigenetic regulation, signaling), CCT2 (cytoskeleton, autophagy, apoptosis), CCT8 (cytoskeleton, autophagy, apoptosis), TCP1 (cytoskeleton, autophagy, apoptosis), RPL7A (carcinogenesis), RPL29 (cell surface expression), RPL34 (carcinogenesis)
Previous AML relevance	GNL3, PA2G4

## Data Availability

All raw data and MaxQuant output files from the original cohort can be found at the ProteomeXchange consortium with the dataset identifier PXD014997.

## Supplementary Materials

The following supporting information can be downloaded at: https://www.mdpi.com/article/10.3390/ijms25105080/s1. References [37,38,51,52,57,60,61,62,63,64,65,66,67,68,69,70,71,72,73,74,75,76,77,78,79,80,81,82,83,84,85,127] are cited in the supplementary materials.
