# Supplementary material for: Monocytic Differentiation of Human Acute Myeloid Leukemia Cells: A Proteomic and Phosphoproteomic Comparison of FAB-M4/M5 Patients with and without Nucleophosmin 1 Mutations
Source: Int J Mol Sci. 2024 May 7;25(10):5080. doi: 10.3390/ijms25105080 (PMC11121526; doi:10.3390/ijms25105080)
Supplement: Supplementary file 1 [file ijms-25-05080-s001.zip › ijms-2809230-supplementary.pdf]

Monocytic differentiation of human acute myeloid leukemia cells: a proteomic and phosphoproteomic comparison of FAB-M4/M5 patients with and without nucleophosmin-1 mutations

Frode Selheim, Elise Aasebø, Håkon Reikvam, Øystein Bruserud and Maria Hernandez-Valladares

Table S1: Clinical and biological characteristics of the 25 patients included in the study; **Table S2**: Proteins showing significantly increased levels in *NPM1-Ins* patients compared with patients without *NPM1-Ins*; **Table S3**: The proteomic comparison of FAB-M4/M5 AML cells with and without *NPM1-Ins*; a summary of proteins forming interacting networks and showing increased levels in patients with *NPM1-Ins*; **Table S4**: Proteins showing significantly increased levels in patients without *NPM1-Ins* compared with patients with *NPM1-Ins*; **Table S5**: The proteomic comparison of FAB-M4/M5 AML cells with and without *NPM1-Ins*; a summary of proteins forming interacting networks and showing increased levels in patients without *NPM1-Ins*; **Table S6**: Protein phosphorylation sites showing significantly increased levels in patients with *NPM1-Ins* compared with patients without *NPM1-Ins*; **Table S7**: The phosphoproteomic comparison of FAB-M4/M5 AML cells with and without *NPM1-Ins*; a summary of proteins forming interacting networks and showing increased phosphorylation levels in patients with (spliceasome, proteasome) and without (ribosome biogenesis, NuRD complex) *NPM1-Ins*; **Table S8**: Protein phosphorylation sites showing significantly increased levels in patients without *NPM1-Ins* compared with patients with *NPM1-Ins*; **Figure S1:** Molecular genetic analyses of primary AML cells with and without NPM1-Ins; **Figure S2**: Hierarchical clustering analysis including all our FAB-M4/M5 patients and based on ribosomal proteins included in identified protein-protein interaction (PPI) networks; **Figure S3:** Principal component (**a**) and unsupervised hierarchical clustering (**b**) analysis of FAB-M4/M5 patients with and without (referred to as *NPM1-wt* in the figure) *NPM1-Ins;* a comparison with normal $CD34^+$ bone marrow cells (referred to as healthy controls in the figure); **Figure S4:** Principal component (**a**) and unsupervised hierarchical clustering (**b**) analysis of FAB-M4/M5 patients with and without (referred to as *NPM1-wt* in the figure) *NPM1-Ins;* a comparison with normal $CD34^+$ bone marrow cells (referred to as healthy controls in the figure).

Table S1. Clinical and biological characteristics of 25 patients included in the study. The table presents the patient identity, gender (M, male; F, female), patients with secondary AML (MDS, myelodysplastic syndrome), bone marrow blasts (percent of nucleated cells), peripheral blood blast count (x 10^9/L) and genetic abnormalities. (wt, wildtype) and time to/course after relapse.

Study ID	Gender	Age (years)	Secondary	BM blasts	Blood blast count	Karyotype	Flt3	NPM1	Months from diagnosis to relapse	Clinical course after relase
P2	M	29		90	18.6	Normal	ITD	Ins	12	Death from relapse
P13	F	45	Chemotherapy	75	22.1	Normal	wt	Ins		
P14	F	42		86	40.4	inv(16)(q13q22)	wt	Ins		
P18	F	59	MDS	83	57.8	Normal	ITD	Ins	8	Death from relapse
P23	M	46		97	170.0	Normal	ITD	Ins	11	Death from relapse
P24	M	53		50	31.4	del(9)(q13q33)	wt	Ins		
P34	M	48		76	14.3	Normal	ITD	Ins	6	Death from relapse
P44	M	65		77	101.0	Normal	wt	Ins		
P46	F	60		83	36.9	Normal	ITD	Ins		
P47	F	41		55	8.6	Normal	wt	Ins		
P49	M	54	Chemotherapy	80	29.1	Normal	wt	Ins		
P51	F	32		95	112.0	t(9;11)(p21;q23)	wt	Ins	18	Long-term survival after allotransplantation
P53	M	48		50	65.3	Normal	wt	Ins		
P10	M	57		89	10.8	46,XY,inv(16)(p13q22)	wt	wt		
P11	F	18		40	33.8	inv(16)(13;q22)/46,idem, t(X;6)(p22;p12)[/46,XX	wt	wt	11	Long-term survival after allotransplantation
P17	M	60		53	14.2	inv(16)	ITD	wt	6	Death from relapse
P22	M	41		30	3.8	inv(16)(p13q22)	wt	wt	26	Death from relapse
P29	M	58		83	70.7	Multiple	wt	wt		
P31	F	36	Chemotherapy	86	37.6	Normal	wt	wt		
P36	M	36		32	33.1	Normal	wt	wt		
P37	F	57		47	65.4	Normal	wt	wt	12	Death from relapse
P42	M	43		90	351.0	Normal	wt	wt		
P43	M	48		52	50.1	del(5)(q31q34)[5]/46,XX[15]	wt	wt		
P45	F	61	MDS	95	24.1	+8	ITD	wt	12	Long-term survival after allotransplantation
P52	M	62		25	33.3	Normal	wt	wt	8	Death from relapse

Table S2. Proteins showing significantly increased levels in *NPM1-Ins* patients compared with patients without *NPM1-Ins*. A total of 217 proteins were identified, and the table presents the gene name, the protein name and the p-value from ANOVA analysis. The proteins are ranged according to their p-value. Proteins also identified in protein-protein interaction (PPI) analyses are marked with grey shadow.

Gene name	Protein name	ANOVA p-value
AK2	Adenylate kinase 2, mitochondrial	1.25E-07
LNPEP	Leucyl-cystinyl aminopeptidase	6.28E-07
PIGK	GPI-anchor transamidase	8.66E-07
NCEH1	Neutral cholesterol ester hydrolase 1	1.19E-06
DMXL2	DmX-like protein 2	3.60E-06
COMMD5	COMM domain-containing protein 5	4.60E-06
C17orf59	Uncharacterized protein C17orf59	6.33E-06
HSD17B4	Peroxisomal multifunctional enzyme type 2	7.28E-06
FDXR	NADPH:adrenodoxin oxidoreductase, mitochondrial	1.21E-05
EMD	Emerin	160E-05
OSBPL8	Oxysterol-binding protein-related protein 8	2.63E-05
PIGS	GPI transamidase component PIG-S	2.67E-05
PIGT	GPI transamidase component PIG-T	3.03E-05
RAB3D	Ras-related protein Rab-3D	3.92E-05
LPCAT3	Lysophospholipid acyltransferase 5	3.93E-05
RAB4B	Ras-related protein Rab-4B	4.36E-05
EVI2B	Protein EVI2B	4.53E-05
EPB41L3	Band 4.1-like protein 3	5.86E-05
C10orf54	Platelet receptor Gi24	6.15E-05
PARP4	Poly [ADP-ribose] polymerase 4	6.85E-05
MAN1B1	Endoplasmic reticulum mannosyl-oligosaccharide 1,2-alpha-mannosidase	8.02E-05
VPS18	Vacuolar protein sorting-associated protein 18 homolog	9.05E-05
COMMD9	COMM domain-containing protein 9	9.11E-05
ACSL1	Long-chain-fatty-acid--CoA ligase 1	9.57E-05
TMEM65	Transmembrane protein 65	0.0001
PIGU	Phosphatidylinositol glycan anchor biosynthesis class U protein	0.0001
COMMD10	COMM domain-containing protein 10	0.0001
GPAA1	Glycosylphosphatidylinositol anchor attachment 1 protein	0.0001
SGPL1	Sphingosine-1-phosphate lyase 1	0.0001
CFD	Complement factor D	0.0001
ADPGK	ADP-dependent glucokinase	0.0002
METTL7B	Methyltransferase-like protein 7B	0.0002
HSDL2	Hydroxysteroid dehydrogenase-like protein 2	0.0002
TMEM192	Transmembrane protein 192	0.0002
GGT1;GGT3P;GGT2	Gamma-glutamyltranspeptidase 1	0.0002
FCGR1A	High affinity immunoglobulin gamma Fc receptor I	0.0002
CCDC22	Coiled-coil domain-containing protein 22	0.0002
CD97	CD97 antigen subunit alpha/ beta	0.0002
ATP11A	Probable phospholipid-transporting ATPase IH	0.0003
FAM101B	Protein FAM101B	0.0003
NUFIP2	Nuclear fragile X mental retardation-interacting protein 2	0.0003

B3GAT3	Galactosylgalactosylxylosylprotein 3-beta-glucuronosyltransferase 3	0.0003
DNASE1L1	Deoxyribonuclease-1-like 1	0.0004
IGF2BP2	Insulin-like growth factor 2 mRNA-binding protein 2	0.0004
VAPA	Vesicle-associated membrane protein-associated protein A	0.0004
ARMCX3	Armadillo repeat-containing X-linked protein 3	0.0004
HOMER3	Homer protein homolog 3	0.0005
ERLIN1	Erlin-1	0.0005
LMAN2	Vesicular integral-membrane protein VIP36	0.0005
IGF2R	Cation-independent mannose-6-phosphate receptor	0.0005
UGGT1	UDP-glucose:glycoprotein glucosyltransferase 1	0.0005
IGF2BP3	Insulin-like growth factor 2 mRNA-binding protein 3	0.0005
RAB24	Ras-related protein Rab-24	0.0005
ARL6IP5	PRA1 family protein 3	0.0005
AGPAT3	1-acyl-sn-glycerol-3-phosphate acyltransferase gamma	0.0005
SLC38A10	Putative sodium-coupled neutral amino acid transporter 10	0.0005
MTCH1	Mitochondrial carrier homolog 1	0.0005
LACTB	Serine beta-lactamase-like protein LACTB, mitochondrial	0.0006
TM9SF4	Transmembrane 9 superfamily member 4	0.0006
ETFA	Electron transfer flavoprotein subunit alpha, mitochondrial	0.0006
FAM45A;FAM45B	Protein FAM45A;Protein FAM45B	0.0006
KPNA4	Importin subunit alpha-3	0.0007
C16orf62	UPF0505 protein C16orf62	0.0007
GHDC	GH3 domain-containing protein	0.0007
ETFB	Electron transfer flavoprotein subunit beta	0.0008
MTDH	Protein LYRIC	0.0008
PLBD2	Putative phospholipase B-like 2	0.0008
ABHD16A	Alpha/beta hydrolase domain-containing protein 16A	0.0009
FAM134C	Protein FAM134C	0.0010
ASCC2	Activating signal cointegrator 1 complex subunit 2	0.0010
CPNE8	Copine-8	0.0010
HSF1	Heat shock factor protein 1	0.0010
STX8	Syntaxin-8	0.0011
LAIR1	Leukocyte-associated immunoglobulin-like receptor 1	0.0011
ADCY7	Adenylate cyclase type 7	0.0011
RNASE2	Non-secretory ribonuclease	0.0012
POFUT1	GDP-fucose protein O-fucosyltransferase 1	0.0012
UBE2J1	Ubiquitin-conjugating enzyme E2 J1	0.0012
BCL2L13	Bcl-2-like protein 13	0.0013
CTSA	Lysosomal protective protein	0.0014
FNDC3B	Fibronectin type III domain-containing protein 3B	0.0014
CLINT1	Clathrin interactor 1	0.0014
NFKB2	Nuclear factor NF-kappa-B p100 subunit	0.0015
PLAUR	Urokinase plasminogen activator surface receptor	0.0015
ABHD11	Alpha/beta hydrolase domain-containing protein 11	0.0016
WDFY1	WD repeat and FYVE domain-containing protein 1	0.0016
ACADVL	Very long-chain specific acyl-CoA dehydrogenase, mitochondrial	0.0017

LNP	Protein lunapark	0.0017
SP110	Sp110 nuclear body protein	0.0018
KIAA1033	WASH complex subunit 7	0.0019
AFTPH	Aftiphilin	0.0019
CRELD1	Cysteine-rich with EGF-like domain protein 1	0.0019
HEATR3	HEAT repeat-containing protein 3	0.0020
JMJD6	Bifunctional arginine demethylase and lysyl-hydroxylase JMJD6	0.0020
DNAJC1	DnaJ homolog subfamily C member 1	0.0020
DCXR	L-xylulose reductase	0.0022
SLC39A11	Zinc transporter ZIP11	0.0022
CCDC93	Coiled-coil domain-containing protein 93	0.0023
OSBPL5	Oxysterol-binding protein-related protein 5	0.0024
PLA2G4A	Cytosolic phospholipase A2	0.0024
PNPLA6	Neuropathy target esterase	0.0024
ASCC3	Activating signal cointegrator 1 complex subunit 3	0.0025
CLTB	Clathrin light chain B	0.0025
SEC22B	Vesicle-trafficking protein SEC22b	0.0025
MYOF	Myoferlin	0.0027
COMMD3	COMM domain-containing protein 3	0.0027
UFSP2	Ufm1-specific protease 2	0.0027
STAB1	Stabilin-1	0.0028
ADAM17	Disintegrin and metalloproteinase domain-containing protein 17	0.0028
PLEC	Plectin	0.0029
DYNC1LI2	Cytoplasmic dynein 1 light intermediate chain 2	0.0030
SPCS1	Signal peptidase complex subunit 1	0.0031
TRIP12	E3 ubiquitin-protein ligase TRIP12	0.0031
DHRS9	Dehydrogenase/reductase SDR family member 9	0.0031
DHRS7B	Dehydrogenase/reductase SDR family member 7B	0.0033
CAT	Catalase	0.0033
DNAJC10	DnaJ homolog subfamily C member 10	0.0037
SFXN3	Sideroflexin-3	0.0034
STX12	Syntaxin-12	0.0034
VAMP7	Vesicle-associated membrane protein 7	0.0034
TPP1	Tripeptidyl-peptidase 1	0.0035
CISD3	CDGSH iron-sulfur domain-containing protein 3, mitochondrial	0.0036
ABCD3	ATP-binding cassette sub-family D member 3	0.0036
STAU1	Double-stranded RNA-binding protein Staufen homolog 1	0.0037
RRBP1	Ribosome-binding protein 1	0.0037
TMEM205	Transmembrane protein 205	0.0038
LAMTOR4	Ragulator complex protein LAMTOR4	0.0038
LMNA	Prelamin-A/C; Lamin-A/C	0.0039
CCDC6	Coiled-coil domain-containing protein 6	0.0039
PTPLAD2	Very-long-chain (3R)-3-hydroxyacyl-CoA dehydratase 4	0.0039
RETSAT	All-trans-retinol 13,14-reductase	0.0042
FAM21A	WASH complex subunit FAM21A	0.0043
TLR2	Toll-like receptor 2	0.0044

TOLLIP	Toll-interacting protein	0.0048
LAMTOR2	Ragulator complex protein LAMTOR2	0.0048
STIM1	Stromal interaction molecule 1	0.0049
GLB1	Beta-galactosidase	0.0049
CTSD	Cathepsin D light/heavy chain	0.0050
HK1	Hexokinase-1	0.0051
BASP1	Brain acid soluble protein 1	0.0052
VPS8	Vacuolar protein sorting-associated protein 8 homolog	0.0056
SPR	Sepiapterin reductase	0.0057
PRCP	Lysosomal Pro-X carboxypeptidase	0.0057
VIM	Vimentin	0.0058
ERLIN2	Erlin-2	0.0058
	Uncharacterized protein FLJ45252	0.0059
MFSD10	Major facilitator superfamily domain-containing protein 10	0.0059
TBL2	Transducin beta-like protein 2	0.0061
COMMD4	COMM domain-containing protein 4	0.0062
SOD2	Superoxide dismutase [Mn], mitochondrial	0.0063
RFT1	Protein RFT1 homolog	0.0070
BST2	Bone marrow stromal antigen 2	0.0071
RPS6KA5	Ribosomal protein S6 kinase alpha-5	0.0071
SLC35A1	CMP-sialic acid transporter	0.0071
RNF170	E3 ubiquitin-protein ligase RNF170	0.0071
CARD9	Caspase recruitment domain-containing protein 9	0.0072
VAMP8	Vesicle-associated membrane protein 8	0.0077
MGST2	Microsomal glutathione S-transferase 2	0.0079
GLA	Alpha-galactosidase A	0.0079
STK11IP	Serine/threonine-protein kinase 11-interacting protein	0.0079
TMED7 (TICAM2)	Transmembrane emp24 domain-containing protein 7	0.0079
DSCR3	Down syndrome critical region protein 3	0.0080
CPVL	Probable serine carboxypeptidase CPVL	0.0086
GPD1L	Glycerol-3-phosphate dehydrogenase 1-like protein	0.0089
OTUD4	OTU domain-containing protein 4	0.0092
DNAJC3	DnaJ homolog subfamily C member 3	0.0096
COMMD8	COMM domain-containing protein 8	0.0096
PHKA2	Phosphorylase b kinase regulatory subunit alpha, liver isoform	0.0101
SELENOF	15 kDa selenoprotein	0.0103
BLOC1S2	Biogenesis of lysosome-related organelles complex 1 subunit 2	0.0105
BCKDK	[3-methyl-2-oxobutanoate dehydrogenase [lipoamide]] kinase, mitochondrial	0.0105
SLC44A1	Choline transporter-like protein 1	0.0107
MVP	Major vault protein	0.0108
VPS16	Vacuolar protein sorting-associated protein 16 homolog	0.0110
DTYMK	Thymidylate kinase	0.0115
CC2D1B	Coiled-coil and C2 domain-containing protein 1B	0.0116
WDR11	WD repeat-containing protein 11	0.0122
SCARB2	Lysosome membrane protein 2	0.0123
PRKCH	Protein kinase C eta type	0.0129

EIF2AK4	Eukaryotic translation initiation factor 2-alpha kinase 4	0.0132
SRGN	Serglycin	0.0136
ALDH3B1	Aldehyde dehydrogenase family 3 member B1	0.0136
DPP7	Dipeptidyl peptidase 2	0.0140
ZBTB7B	Zinc finger and BTB domain-containing protein 7B	0.0142
TOR1AIP1	Torsin-1A-interacting protein 1	0.0145
AGTRAP	Type-1 angiotensin II receptor-associated protein	0.0147
USP32	Ubiquitin carboxyl-terminal hydrolase 32	0.0148
STX16	Syntaxin-16	0.0148
ERLEC1	Endoplasmic reticulum lectin 1	0.0148
PTPN9	Tyrosine-protein phosphatase non-receptor type 9	00155
VPS26B	Vacuolar protein sorting-associated protein 26B	0.0158
CMAS	N-acylneuraminate cytidylyltransferase	0.0159
TOM1	Target of Myb protein 1	0.0167
TTYH3	Protein tweety homolog 3	0.0171
VAPB	Vesicle-associated membrane protein-associated protein B/C	0.0172
ERO1L	ERO1-like protein alpha	0.0175
AP3M1	AP-3 complex subunit mu-1	0.0176
VPS11	Vacuolar protein sorting-associated protein 11 homolog	0.0192
CLCN7	H(+)/Cl(-) exchange transporter 7	0.0194
PSTPIP2	Proline-serine-threonine phosphatase-interacting protein 2	0.0202
TOR4A	Torsin-4A	0.0202
SCYL2	SCY1-like protein 2	0.0209
TCIRG1	V-type proton ATPase 116 kDa subunit a isoform 3	0.0210
LEMD2	LEM domain-containing protein 2	0.0238
EHHADH	Peroxisomal bifunctional enzyme;Enoyl-CoA hydratase	0.0240
SEC16A	Protein transport protein Sec16A	0.0248
SCAMP1	Secretory carrier-associated membrane protein 1	0.0248
TMEM175	Transmembrane protein 175	0.0257
AGA	N(4)-(beta-N-acetylglucosaminyl)-L-asparaginase	0.0270
SDF2L1	Stromal cell-derived factor 2-like protein 1	0.0276
CPM	Carboxypeptidase M	0.0276
RAB44	Ras-related protein Rab-44	0.0278
TOR1A	Torsin-1A	0.0296
TRIM4	Tripartite motif-containing protein 4	0.0297
TACC1	Transforming acidic coiled-coil-containing protein 1	0.0301
AP3B1	AP-3 complex subunit beta-1	0,0326
AKAP13	A-kinase anchor protein 13	0.0391

Table S3. The proteomic comparison of FAB-M4/M5 AML cells with and without *NPM1-Ins*; a summary of proteins forming interacting networks (**Figure 1** of the main text) and showing increased levels in patients with *NPM1-Ins*. The table presents the gene name, protein names and key words with regard to protein function. The table is based on information from the Gene database and selected references from the PubMed database (accessed 29th of October 2023) [37,38,51,52,57, 60-64].

**ENDOCYTIC RECYCLING (11 proteins)**		
COMMD3	*COMM domain containing 3.* The encoded protein is predicted to be involved in sodium ion transport, and it is predicted to be located in the extracellular region and ficolin-1-rich granule lumen.	Membrane protein<br>Sodium transport
COMMD4	*COMM domain containing 4.* The encoded protein is located in the cytosol, intracellular membrane-bounded organelles and the plasma membrane.	Cytosole<br>Plasma membrane
COMMD5	*COMM domain containing 4.* The protein I located in cytosol, intracellular membrane-bounded organelles and the plasma membrane.	Cytosole<br>Plasma membrane
COMMD8	*COMM domain containing 8.* The protein binds coiled-coil domain-containing protein 22 (CCDC22), and this complex can regulate the turnover of I-kappa-B and the activation of NF-kappa-B.	NFκB<br>CCDC22 (see below)
COMMD9	*COMM domain containing 9.* The protein is predicted to be involved in sodium ion transport and to act upstream of or within cholesterol homeostasis. It is located in Golgi apparatus, cytosol and nucleoplasm.	Membrane protein<br>Sodium transport<br>Golgi, cytosole, nucleoplasm
COMMD10	*COMM domain containing 10.* Located in nucleoplasm and involved in endosomal recycling.	Nucleoplasm
CCDC22	*Coiled-coil domain containing 22.* This gene encodes a protein containing a coiled-coil domain. The encoded protein functions in the regulation of NFκB by interacting with COMMD (copper metabolism Murr1 domain-containing) proteins. The mouse orthologous protein has been shown to bind copines, which are calcium-dependent, membrane-binding proteins that may function in calcium signaling.	NFκB<br>COMMD protein interactions
CCDC93	*Coiled-coil domain containing 22.* The encoded protein is *i*nvolved in Golgi to plasma membrane transport and endocytic recycling. It is located in intracellular membrane-bounded organelle.	Golgi
VPS26C	*VPS26 endosomal protein sorting factor C.* The protein is important for endosomal transport [37,60,61].	Endosome
VPS35L	*VPS35 endosomal protein sorting factor like.* The encoded protein is involved in Golgi to plasma membrane transport and endocytic recycling. It is located in endosome.	Golgi to plasma membrane<br>Endosome
DENND10	*DENN domain containing 10.* The protein enables guanyl-nucleotide exchange factor activity and small GTPase binding activity. It is involved in endosome transport via the multivesicular body sorting pathway, protein transport and regulation of early endosome to late endosome transport. The protein is thus located in late endosomes.	Endosome<br>Protein transport
**VESICLE FUSION (10 proteins)**		
SEC22B	*SEC22 homolog B, vesicle trafficking protein b.* The encoded protein is one of the SNARE proteins, and it functions in membrane fusion of vesicle trafficking between the endoplasmic reticulum and the Golgi apparatus and in secretory autophagy [38].	SNARE<br>Endoplasmic reticulum<br>Golgi<br>Autophagy
STX8	*Syntaxin 8.* The encoded protein is a member of the syntaxin family and is involved in protein trafficking from early to late endosomes via vesicle fusion and exocytosis. It interacts with the SNARE complex [62-64].	Endosomes<br>Protein trafficking<br>SNARE
STX12	*Syntaxin 12.* The protein is predicted to enable SNAP receptor activity and SNARE binding activity and is involved in autophagosome assembly and protein stabilization. It is located in several cellular components, including membrane rafts, phagocytic vesicles,and phagophore assembly site.	Autophagosome<br>Protein stabilization<br>SNARE
STX16	*Syntaxin 16.* The encoded protein is a member of the syntaxin or t-SNARE (target-SNAP receptor) family. These proteins are found on cell membranes and serve as the targets for V-SNARES (vesicle-SNAP receptors) permitting specific vesicle docking and fusion.	Vesicle docking and fusion<br>SNARE

VAMP7	*Vesicle associated membrane protein 7.* The encoded transmembrane protein that is a member of the soluble N-ethylmaleimide-sensitive factor attachment protein receptor (SNARE) family. It localizes to late endosomes and lysosomes and is involved in the fusion of transport vesicles to their target membranes.	Vesicle fusion<br>SNARE
VAMP8	*Vesicle associated membrane protein 8. The encoded protein is* an integral membrane protein that belongs to the synaptobrevin/vesicle-associated membrane protein subfamily of soluble N-ethylmaleimide-sensitive factor attachment protein receptors (SNAREs). The encoded protein is involved in the fusion of vesicles with the membrane.	Vesicle fusion<br>SNARE
VPS8	*VPS8 subunit of CORVET complex.* The encoded protein is predicted to enable metal ion binding activity. It is involved in endosomal vesicle fusion and located in early endosomes.	Endosome
VPS11	*VPS11 core subunit of CORVET and HOPS complexes.* Vesicle mediated protein sorting plays an important role in segregation of intracellular molecules into distinct organelles. Genetic studies in yeast have identified more than 40 vacuolar protein sorting (VPS) genes involved in vesicle transport to vacuoles. This gene encodes the human homolog of yeast class C Vps11 protein. The mammalian class C Vps proteins are predominantly associated with late endosomes/lysosomes and may mediate vesicle trafficking steps in the endosome/lysosome pathway.	Late endosomes<br>Vesicle trafficking
VPS16	*VPS16 core subunit of CORVET and HOPS complexes.* This gene encodes the human homolog of yeast class C Vps16 protein. The mammalian class C Vps proteins are predominantly associated with late endosomes/lysosomes and may mediate vesicle trafficking steps in the endosome/lysosome pathway.	Late endosome<br>Vesicle trafficking
VPS18	*VPS18 core subunit of CORVET and HOPS complexes.* This gene encodes the human homolog of yeast class C Vps18 protein. The mammalian class C Vps proteins are predominantly associated with late endosomes/lysosomes and mediate vesicle trafficking steps in the endosome/lysosome pathway.	Late endosome<br>Vesicle trafficking
	**THE GLYCOSYLPHOSPHOSPHATIDYLINOSITOL (GPI) TRANSAMIDASE COMPLEX; ATTACHMENT OF THE GPI ANCHOR TO PROTEIN (5 proteins)**	
GPAA1	*Glycosylphosphatidylinositol anchor attachment 1.* Posttranslational glycosylphosphatidylinositol (GPI) anchor attachment serves as a general mechanism for linking proteins to the cell surface membrane. The protein encoded by this gene presumably functions in GPI anchoring at the GPI transfer step. The anchor attachment protein 1 contains an N-terminal signal sequence, 1 cAMP- and cGMP-dependent protein kinase phosphorylation site, 1 leucine zipper pattern, 2 potential N-glycosylation sites, and 8 putative transmembrane domains.<br>These five proteins form the GPI transamidase complex that mediates binding of GPI to target proteins [51,52]. GPI molecules as membrane anchors. GPI-transamidase is a membrane-bound protein complex found in the endoplasmic reticulum; after attachment of GPI to target proteins the lipid reconstitution occurs upon transport to the Golgi [52] . However, GPI anchored proteins are almost exclusively localized to the cell surface where they are associated to the plasma membrane through the lipid portion of the anchor.	GPI anchoring
PIGK	*Phosphatidylinositol glycan anchor biosynthesis class K.* This member of the cysteine protease family C13 is involved in glycosylphosphatidylinositol (GPI)-anchor biosynthesis. The GPI-anchor is a glycolipid that serves to anchor proteins to the cell surface. This protein is a member of the multisubunit enzyme, GPI transamidase and is thought to be its enzymatic component. GPI transamidase mediates GPI anchoring in the endoplasmic reticulum, by catalyzing the transfer of fully assembled GPI units to proteins.	Membrane anchor<br>GPI transamidase<br>Endoplasmic reticulum
PIGS	*Phosphatidylinositol glycan anchor biosynthesis class S.* The encoded protein is involved in GPI-anchor biosynthesis. The glycosylphosphatidylinositol (GPI) anchor is a glycolipid serves to anchor proteins to the cell surface. This gene encodes an essential component of the multisubunit enzyme GPI transamidase that mediates GPI anchoring in the endoplasmic reticulum by catalyzing the transfer of fully assembled GPI units to proteins.	Membrane anchor<br>GPI transamidase<br>Endoplasmic reticulum
PIGT	*Phosphatidylinositol glycan anchor biosynthesis class T.* This gene encodes a protein involved in glycosylphosphatidylinositol (GPI)-anchor biosynthesis. This protein is an essential component of the multisubunit enzyme, GPI transamidase that mediates GPI anchoring in the endoplasmic reticulum, by catalyzing the transfer of fully assembled GPI units to proteins.	Membrane anchor<br>GPI transamidase<br>Endoplasmic reticulum
PIGU	*Phosphatidylinositol glycan anchor biosynthesis class U.* The encoded protein is a predicted integral membrane protein that may function in cell division control. It is the fifth subunit of GPI transamidase that attaches GPI-anchors to proteins.	Membrane anchor<br>GPI transamidase<br>Endoplasmic reticulum

	**GALACTOSIDASE ACTIVITY (4 proteins)**	
HK1	*Hexokinase 1.* Hexokinases phosphorylate glucose to produce glucose-6-phosphate, the first step in most glucose metabolism pathways. This gene encodes a ubiquitous form of hexokinase which localizes to the outer membrane of mitochondria.	Mitochondria<br>Glucose metabolism
GLB1	*Galactosidase beta 1.* This gene encodes a member of the glycosyl hydrolase 35 family of proteins. Alternative splicing results in multiple transcript variants, at least one of which encodes a preproprotein that is proteolytically processed to generate the mature lysosomal enzyme. This enzyme catalyzes the hydrolysis of a terminal beta-linked galactose residue from ganglioside substrates and other glycoconjugates.	Lysosomal enzyme
GLA	*Galactosidase alpha.* This gene encodes a homodimeric glycoprotein that hydrolyses the terminal alpha-galactosyl moieties from glycolipids and glycoproteins. This enzyme predominantly hydrolyzes ceramide trihexoside, and it can catalyze the hydrolysis of melibiose into galactose and glucose. A variety of mutations in this gene affect the synthesis, processing, and stability of this enzyme, which causes Fabry disease, a rare lysosomal storage disorder that results from a failure to catabolize alpha-D-galactosyl glycolipid moieties.	Lysosomal enzyme
CTSA	*Cathepsin A.* This gene encodes a member of the peptidase S10 family of serine carboxypeptidases. Alternative splicing results in multiple transcript variants, at least one of which encodes a preproprotein that is proteolytically processed to generate two chains that comprise the heterodimeric active enzyme. This enzyme possesses deamidase, esterase and carboxypeptidase activities and acts as a scaffold in the lysosomal multienzyme complex.	Lysosome
	**CLATHRIN BINDING (3 proteins)**	
CLINT1	*Clatrin interactor 1.* The encoded protein has similarity to the epsin family of endocytic adapter proteins. It interacts with clathrin, the adapter protein AP-1 and phosphoinositides. This protein may be involved in the formation of clathrin coated vesicles and trafficking between the trans-Golgi network and endosomes.	Clathrin-coated vesicles<br>Endosome<br>Trans-Golgi network
CLTB	*Clathrin light chain beta.* Clathrin is a large, soluble protein composed of heavy and light chains. It functions as the main structural component of the lattice-type cytoplasmic face of coated pits and vesicles which entrap specific macromolecules during receptor-mediated endocytosis. This gene encodes one of two clathrin light chain proteins which are believed to function as regulatory elements.	Clathrin-mediated and receptor-mediated endocytosis
AFTPH	*Aftiphilin.* The encoded protein enables clathrin binding activity and is predicted to be involved in intracellular transport. It is located in the Golgi apparatus, cytosol and nucleoplasm, and it is a part of AP-1 adaptor protein complex. It can also bind to the clathrin adaptor protein AP-2 that is regarded as a key component in clathrin-mediated endocytosis [57].	Intracellular transport<br>Golgi, cytosol, nucleoplasm

Table S4. Proteins showing significantly increased levels in patients without *NPM1-Ins* compared with patients with *NPM1-Ins*. A total of 132 proteins were identified, and the table presents the gene name, the protein name and the p-value from ANOVA analysis. The proteins are ranged according to their p-value. Proteins also identified in PPI analyses are marked with grey shadow.

Gene name	Protein name	ANOVA p-value
SRP14	Signal recognition particle 14 kDa protein	4.10E-08
SRP9	Signal recognition particle 9 kDa protein	1.05E-07
GNG2	Guanine nucleotide-binding protein G(I)/G(S)/G(O) subunit gamma-2	9.67E-06
NPM1	Nucleophosmin	1.16E-05
NLE1	Notchless protein homolog 1	4.22E-05
RRM1	Ribonucleoside-diphosphate reductase large subunit	4.70E-05
NO66	Bifunctional lysine-specific demethylase and histidyl-hydroxylase NO66	4.76E-05
RPL15	60S ribosomal protein L15	5.28E-05
OGT	UDP-N-acetylglucosamine--peptide N-acetylglucosaminyltransferase 110 kDa subunit	8.07E-05
MEF2D	Myocyte-specific enhancer factor 2D	8.43E-05
PAICS	Multifunctional protein ADE2	0.0002
YBX1	Nuclease-sensitive element-binding protein 1	0.0002
RPL12	60S ribosomal protein L12	0.0004
RAD23B	UV excision repair protein RAD23 homolog B	0.0004
PA2G4	Proliferation-associated protein 2G4	0.0004
RPL29	60S ribosomal protein L29	0.0004
NACA	Nascent polypeptide-associated complex subunit alpha	0.0004
RPL19	60S ribosomal protein L19	0.0005
PLD4	Phospholipase D4	0.0006
NPM3	Nucleoplasmin-3	0.0007
RPL34	60S ribosomal protein L34	0.0008
RPS9	40S ribosomal protein S9	0.0008
SERPINB9	Serpin B9	0.0008
EIF5B	Eukaryotic translation initiation factor 5B	0.0009
CLIC4	Chloride intracellular channel protein 4	0.0011
RPL13	60S ribosomal protein L13	0.0011
MAD2L1	Mitotic spindle assembly checkpoint protein MAD2A	0.0011
UBA5	Ubiquitin-like modifier-activating enzyme 5	0.0013
PM20D2	Peptidase M20 domain-containing protein 2	0.0013
EEF2	Elongation factor 2	0.0014
NAP1L4	Nucleosome assembly protein 1-like 4	0.0015
RPL32	60S ribosomal protein L32	0.0016
ENY2	Transcription and mRNA export factor ENY2	0.0017
FTSJ3	pre-rRNA processing protein FTSJ3	0.0017
SERBP1	Plasminogen activator inhibitor 1 RNA-binding protein	0.0017
UBQLN2	Ubiquilin-2	0.0019
RPL4	60S ribosomal protein L4	0.0019
TXNL1	Thioredoxin-like protein 1	0.0021
RPL13A	60S ribosomal protein L13a	0.0022
MTHFD1	C-1-tetrahydrofolate synthase, cytoplasmic	0.0022
CBX5	Chromobox protein homolog 5	0.0023

IPO5	Importin-5	0.0026
UTP3	Something about silencing protein 10	0.0027
METAP2	Methionine aminopeptidase 2	0.0029
RPL14	60S ribosomal protein L14	0.0029
PFAS	Phosphoribosylformylglycinamidine synthase	0.0029
RPL36	60S ribosomal protein L36	0.0031
HAX1	HCLS1-associated protein X-1	0.0032
RPL18A	60S ribosomal protein L18a	0.0032
MPO	Myeloperoxidase	0.0032
RPS28	40S ribosomal protein S28	0.0034
RPL7	60S ribosomal protein L7	0.0034
RPS5	40S ribosomal protein S5	0.0035
FAF1	FAS-associated factor 1	0.0035
DDX54	ATP-dependent RNA helicase DDX54	0.0037
SLK	STE20-like serine/threonine-protein kinase	0.0037
CCT2	T-complex protein 1 subunit beta	0.0040
RPL18	60S ribosomal protein L18	0.0043
SAMD9	Sterile alpha motif domain-containing protein 9	0.0045
SGTA	Small glutamine-rich tetratricopeptide repeat-containing protein alpha	0.0046
BMS1	Ribosome biogenesis protein BMS1 homolog	0.0049
KRR1	KRR1 small subunit processome component homolog	0.0051
ASF1A	Histone chaperone ASF1A	0.0054
RPL27A	60S ribosomal protein L27a	0.0055
IPO4	Importin-4	0.0056
CDK1	Cyclin-dependent kinase 1	0.0056
NOC3L	Nucleolar complex protein 3 homolog	0.0056
TCP1	T-complex protein 1 subunit alpha	0.0057
NLRX1	NLR family member X1	0.0058
RCL1	RNA 3-terminal phosphate cyclase-like protein	0.0059
NUDT5	ADP-sugar pyrophosphatase	0.0059
ZCCHC11	Terminal uridylyltransferase 4	0.0061
CCT8	T-complex protein 1 subunit theta	0.0062
RRS1	Ribosome biogenesis regulatory protein homolog	0.0062
RPL26	60S ribosomal protein L26	0.0066
RPL21	60S ribosomal protein L21	0.0067
UBFD1	Ubiquitin domain-containing protein UBFD1	0.0068
RPL23A	60S ribosomal protein L23a	0.0070
IRGQ	Immunity-related GTPase family Q protein	0.0072
GID8	Glucose-induced degradation protein 8 homolog	0.0074
GALK2	N-acetylgalactosamine kinase	0.0075
RPL28	60S ribosomal protein L28	0.0079
STMN1	Stathmin	0.0082
PFDN2	Prefoldin subunit 2	0.0084
DCAF8	DDB1- and CUL4-associated factor 8	0.0087
RPL31	60S ribosomal protein L31	0.0089
GNL3	Guanine nucleotide-binding protein-like 3	0.0090

SERPINH1	Serpin H1	0.0091
UBE2A	Ubiquitin-conjugating enzyme E2 A	0.0094
RPL11	60S ribosomal protein L11	0.0095
EBNA1BP2	Probable rRNA-processing protein EBP2	0.0098
CMPK2	UMP-CMP kinase 2, mitochondrial	0.0098
SAAL1	Protein SAAL1	0.0099
NAA15	N-alpha-acetyltransferase 15, NatA auxiliary subunit	0.0101
RPL7A	60S ribosomal protein L7a	0.0106
DNMT3A	DNA (cytosine-5)-methyltransferase 3A	0.0109
RBM3	Putative RNA-binding protein 3	0.0110
PDCD5	Programmed cell death protein 5	0.0113
FSCN1	Fascin	0.0113
RPIA	Ribose-5-phosphate isomerase	0.0113
MYO18A	Unconventional myosin-XVIIIa	0.0113
RPS20	40S ribosomal protein S20	0.0117
RPL3	60S ribosomal protein L3	0.0119
FKBP4	Peptidyl-prolyl cis-trans isomerase FKBP4;	0.0122
RPS17	40S ribosomal protein S17	0.0123
PATL1	Protein PAT1 homolog 1	0.0123
RPL24	60S ribosomal protein L24	0.0125
BCAT1	Branched-chain-amino-acid aminotransferase, cytosolic	0.0127
CTPS1	CTP synthase 1	0.0130
BTF3	Transcription factor BTF3	0.0138
RPS24	40S ribosomal protein S24	0.0139
RPS29	40S ribosomal protein S29	0.0143
RPL9	60S ribosomal protein L9	0.0147
RPS18	40S ribosomal protein S18	0.0158
RPLP1	60S acidic ribosomal protein P1	0.0160
RPL36AL	60S ribosomal protein L36a-like	0.0165
PEF1	Peflin	0.0175
RPLP2	60S acidic ribosomal protein P2	0.0180
CCS	Copper chaperone for superoxide dismutase	0.0184
RPS8	40S ribosomal protein S8	0.0194
RPS3	40S ribosomal protein S3	0.0196
DCTD	Deoxycytidylate deaminase	0.0225
UBE2E1	Ubiquitin-conjugating enzyme E2 E1	0.0225
ZC3HAV1L	Zinc finger CCCH-type antiviral protein 1-like	0.0229
HN1L	Hematological and neurological expressed 1-like protein	0.0229
DDX3X	ATP-dependent RNA helicase DDX3X	0.0229
RPS23	40S ribosomal protein S23	0.0237
FAU	40S ribosomal protein S30	0.0277
PCBP2	Poly(rC)-binding protein 2	0.0284
EIF4A1	Eukaryotic initiation factor 4A-I	0.0285
ISYNA1	Inositol-3-phosphate synthase 1	0.0297
LGALS9	Galectin-9	0.0321

Table S5. The proteomic comparison of FAB-M4/M5 AML cells with and without *NPM1-Ins*; a summary of proteins forming interacting networks (**Figure 1** of the main text) and showing increased levels in patients without *NPM1-Ins*. The table presents the gene name, protein names and key words with regard to protein function. The table is based on information from the Gene database and selected references from the PubMed database (accessed 29th of October 2023) [65-76].

CYTOPLASMIC TRANSLATION (61 proteins)

BMS1	*BMS1 ribosome biogenesis factor.* The gene encodes a ribosome assembly protein. A similar protein in yeast functions in 35S-rRNA processing, which includes a series of cleavage steps critical for formation of 40S ribosomes.	Ribosome assembly<br>Ribosome 40S
BTF3	*Basic transcription factor 3*. This gene encodes the basic transcription factor 3. This protein forms a stable complex with RNA polymerase IIB and is required for transcriptional initiation.	Transcription initiation<br>RNA polymerase
EBNAIBP2	*EBNA1 binding protein 2*. The encoded protein enables RNA binding activity. It is predicted to be involved in rRNA processing and ribosomal large subunit biogenesis. The protein is located in chromosome and nucleolus.	RNA binding and processing<br>Nucleolus<br>Ribosome 40S
EEF2	*Eukaryotic translation elongation factor 2*. The encoded member of the GTP-binding translation elongation factor family is essential for protein synthesis. It promotes the GTP-dependent translocation of the nascent protein chain from the A-site to the P-site of the ribosome. The protein is completely inactivated by EF-2 kinase phosporylation.	Translation<br>Elongation<br>Ribosome
EIF4A1	*Eukaryotic translation initiation factor 4A1*. The protein enables double-stranded RNA binding activity; it is predicted to be involved in cytoplasmic translational initiation.	RNA binding<br>Translation initiation
EIF5B	*Eukaryotic translation initiation factor 5B.* Accurate initiation of translation in eukaryotes is complex and requires many factors, some of which are composed of multiple subunits. The encoded protein eIF5B interacts with eIF1A on the ribosome along with other initiation factors and GTP to position the initiation methionine tRNA on the start codon of the mRNA so that translation initiates accurately.	Ribosome<br>Translation initiation
FAU	*FAU ubiquitin like and ribosomal protein S30 fusion*. This gene encodes a fusion protein consisting of the ubiquitin-like protein fubi at the N terminus and ribosomal protein S30 at the C terminus. It has been proposed that the fusion protein is post-translationally processed to generate free fubi and free ribosomal protein S30. Fubi is a member of the ubiquitin family, and ribosomal protein S30 belongs to the S30E family of ribosomal proteins. Ribosomal protein S30 is a component of the 40S subunit of the cytoplasmic ribosome.	Ribosome S40<br>Cytoplasm
FTSJ3	*FtsJ RNA 2'-O-methyltransferase 3*. The gene encodes an RNA methyltransferase; the protein is localized to the nucleolus where it colocalizes with NIP7 and contributes in the formation of preribosomal complexes [70-72].	RNA<br>Nucleolus
GNL3	*G protein nucleolar 3*. The encoded protein may interact with p53, may be involved in carcinogenesis and protein also appears to be important for stem cell proliferation. This protein is found in both the nucleus and nucleolus. GNL3 seems to be involved ribosomal biogenesis as well as in regulation of cell proliferation, resistance to apoptosis, cellular differentiation and aging (for references see [73]. It may also be involved in myeloid leukemogenesis [65].	Ribosome<br>Nucleus, nucleolus<br>Carcinogenesis
KRR1	*KRR1 small subunit processome component homolog*. The protein has RNA binding activity and is predicted to be involved in rRNA processing. It is localized in the nucleus.	RNA binding<br>tRNA processing
NACA	*Nascent polypeptide associated complex subunit alpha.* This encoded protein associates with basic transcription factor 3 (BTF3) to form the nascent polypeptide-associated complex (NAC). This complex binds to nascent proteins that lack a signal peptide motif as they emerge from the ribosome, blocking interaction with the signal recognition particle (SRP) and preventing mistranslocation to the endoplasmic reticulum.	Ribosome-RNA<br>Protein synthesis
NCC3/CCL15	*C-C motif chemokine ligand 15.* This secreted protein is chemotactic for T cells and monocytes, and acts through C-C chemokine receptor type 1 (CCR1). The proprotein is further processed into numerous smaller functional peptides. Naturally-occurring readthrough transcripts occur from this gene into the downstream gene CCL14.	Chemokine CCL15
NLE1	*Notchless homolog 1.* Predicted to be involved in Notch signaling pathway and ribosomal large subunit assembly. Predicted to act in hematopoietic stem cell homeostasis and regulation of signal transduction. Located in nucleolus and nucleoplasm.	Notch signaling<br>Ribosome S60 assembly

PA2G4	*Proliferation associated 2G4.* This RNA-binding protein is present in pre-ribosomal ribonucleoprotein complexes and may be involved in ribosome assembly and the regulation of intermediate and late steps of rRNA processing. This protein can interact with the cytoplasmic domain of the ErbB3 receptor and may contribute to transducing regulatory signals. This protein is also a transcriptional co-repressor of cell cycle regulatory genes through its interactions with histone deacetylases. This protein has been implicated in differentiation of human cancer cells. The protein seems to support leukemogenesis in human AML; Ebp1 is highly expressed in AML cells and regulates the level of ribosomal RNA synthesis by binding to RNA Polymerase I and enhancing the formation of the Pol I initiation complex [66,67]. The protein is also a substrate for MDM2 [66].	Nucleoplasm and nucleolus<br>AML<br>Transcriptional corepressor<br>Histone deacetylation<br>RNA binding<br>Ribosomal assembly<br>Signal transduction<br>Differentiation
RCL1	*RNA terminal phosphate cyclase like 1.* The protein is predicted to enable endoribonuclease activity and to be involved in endonucleolytic cleavage of tricistronic rRNA transcript. Predicted to be located in nucleoplasm and to be active in nucleolus.	Nucleoplasm, nucleolus<br>RNA-endoribomuclease
RPL2	*Ribosomal protein L2.*	Ribosome?
RPL3	*Ribosomal protein L3.* This gene encodes a ribosomal protein that is a component of the 60S subunit. The protein belongs to the L3P family of ribosomal proteins and it is located in the cytoplasm. This gene is co-transcribed with several small nucleolar RNA genes.	Ribosome 60S<br>Cytoplasm
RPL4	*Ribosomal protein L4.* This ribosomal protein is a component of the 60S subunit and belongs to the L4E family of ribosomal proteins. It is located in the cytoplasm.	Ribosome 60S<br>Cytoplasm
RPL7	*Ribosomal protein L7.* The encoded ribosomal protein is a component of the 60S subunit and belongs to the L30P family of ribosomal proteins. It contains an N-terminal basic region-leucine zipper (BZIP)-like domain and the RNP consensus submotif RNP2. In vitro the BZIP-like domain mediates homodimerization and stable binding to DNA and RNA, with a preference for 28S rRNA and mRNA. The protein can inhibit cell-free translation of mRNAs, suggesting that it plays a regulatory role in the translation apparatus. It is located in the cytoplasm.	Ribosome 60S<br>Cytoplasm<br>RNA binding<br>DNA binding<br>Inhibition of translation?
RPL7A	*Ribosomal protein L7A.* This gene encodes a ribosomal protein that is a component of the 60S subunit. The protein belongs to the L7AE family of ribosomal proteins. It can interact with a subclass of nuclear hormone receptors and inhibit their ability to transactivate by preventing their binding to their DNA response elements. The gene is co-transcribed with the U24, U36a, U36b, and U36c small nucleolar RNA genes. This gene may be involved in carcinogenesis; it rearranges with the trk proto-oncogene to form the chimeric oncogene trk-2h, which encodes an oncoprotein consisting of the N terminus of ribosomal protein L7a fused to the receptor tyrosine kinase domain of trk [68,69].	Ribosome 60S<br>Nuclear receptor inhibition<br>Chimeric oncoprotein
RPL9	*Ribosomal protein P1.* This gene encodes a ribosomal protein that is a component of the 60S subunit. The protein belongs to the L6P family of ribosomal proteins. It is located in the cytoplasm.	Ribosome 60S<br>Cytoplasm
RPL11	*Ribosomal protein L11.* Ribosomes consist of a small 40S subunit and a large 60S subunit. Together these subunits are composed of 4 RNA species and approximately 80 structurally distinct proteins. This gene encodes a ribosomal protein that is a component of the 60S subunit. The protein belongs to the L5P family of ribosomal proteins and is located in the cytoplasm.	Ribosome 60S<br>Cytoplasm
RPL12	*Ribosomal protein L12.* This gene encodes a component of the 60S ribosomal subunit. It belongs to the L11P family of ribosomal proteins and is located in the cytoplasm. The protein binds directly to the 26S rRNA. This gene is co-transcribed with the U65 snoRNA.	Ribosome 60S<br>Cytoplasm
RPL13	*Ribosomal protein L13.* The encoded ribosomal protein is a component of the 60S subunit. It belongs to the L13E family of ribosomal proteins and is located in the cytoplasm.	Ribosome 60S<br>Cytoplasm
RPL14	*Ribosomal protein L14.* This ribosomal protein is a component of the 60S subunit. It belongs to the L14E family of ribosomal proteins and is located in the cytoplasm.	Ribosome 60S<br>Cytoplasm
RPL15	*Ribosomal protein L15.* This protein is a member of the L15E family of ribosomal proteins and a component of the 60S subunit.	Ribosome 60S
RPL18	*Ribosomal protein L18.* This protein is a member of the L18E family of ribosomal proteins and is a component of the 60S subunit.	Ribosome 60S
RPL18A	*Ribosomal protein L18A.* This gene encodes a member of the L18AE family of ribosomal proteins and is a component of the 60S subunit. This gene is co-transcribed with the U68 snoRNA, located within the third intron.	Ribosome 60S
RPL19	*Ribosomal protein L19.* This gene encodes a ribosomal protein that is a component of the 60S subunit. The protein belongs to the L19E family of ribosomal proteins. It is located in the cytoplasm.	Ribosome 60S<br>Cytoplasm

RPL21	*Ribosomal protein L21.* This gene encodes a ribosomal protein that is a component of the 60S subunit. It belongs to the L21E family of ribosomal proteins and is located in the cytoplasm.	Ribosome 60S<br>Cytoplasm
RPL23A	*Ribosomal protein L23A.* This gene encodes a ribosomal protein that is a component of the 60S subunit. It belongs to the L23P family of ribosomal proteins and is located in the cytoplasm. This gene is co-transcribed with the U42A, U42B, U101A, and U101B small nucleolar RNA genes, which are located in its third, first, second, and fourth introns, respectively.	Ribosome 60S<br>Cytoplasm
RPL24	*Ribosomal protein L24.* The encoded ribosomal protein is a component of the 60S subunit. It belongs to the L24E family of ribosomal proteins and is located in the cytoplasm.	Ribosome 60S<br>Cytoplasm
RPL26	*Ribosomal protein L26.* This encoded ribosomal protein is a component of the 60S subunit. The protein belongs to the L24P family of ribosomal proteins. It is located in the cytoplasm.	Ribosome 60S<br>Cytoplasm
RPL27A	*Ribosomal protein L27A.* This gene encodes a ribosomal protein that is a component of the 60S subunit. It belongs to the L15P family of ribosomal proteins and is located in the cytoplasm.	Ribosome 60S<br>Cytoplasm
RPL28	*Ribosomal protein L28.* This gene encodes a ribosomal protein that is a component of the 60S subunit. The protein belongs to the L28E family of ribosomal proteins and is located in the cytoplasm.	Ribosome 60S<br>Cytoplasm
RPL29	*Ribosomal protein L29.* The gene encodes a cytoplasmic ribosomal protein that is a component of the 60S subunit. The protein belongs to the L29E family of ribosomal proteins. The protein is also a peripheral membrane protein expressed on the cell surface.	Ribosome 60S<br>Cytoplasm<br>Cell surface
RPL31	*Ribosomal protein L31.* This gene encodes a ribosomal protein that is a component of the 60S subunit. The protein belongs to the L31E family of ribosomal proteins and is located in the cytoplasm.	Ribosome 60S<br>Cytoplasm
RPL32	*Ribosomal protein L32.* This gene encodes a ribosomal protein that is a component of the 60S subunit. It belongs to the L32E family of ribosomal proteins and is located in the cytoplasm.	Ribosome 60S<br>Cytoplasm
RPL34	*Ribosomal protein L34.* Th encoded ribosomal protein is a component of the 60S subunit and belongs to the L34E family of ribosomal proteins. It is located in the cytoplasm. Overexpression of this gene has been observed in some cancer cells.	Ribosome 60S<br>Cytoplasm<br>Cancer
RPL36	*Ribosomal protein L36.* The encoded ribosomal protein is a component of the 60S subunit and protein belongs to the L36E family of ribosomal proteins. It is located in the cytoplasm.	Ribosome 60S<br>Cytoplasm
RPL36AL	*Ribosomal protein L36A like.* This gene encodes a ribosomal protein that is a component of the 60S subunit and belongs to the L44E (L36AE) family of ribosomal proteins.	Ribosome 60S
RPLP1	*Ribosomal protein lateral stalk subunit P1.* This encoded ribosomal phosphoprotein is a component of the 60S subunit, belongs to the L12P family of ribosomal proteins and has an important role in the elongation step of protein synthesis. The protein is located in the cytoplasm.	Ribosome 60S<br>Protein elongation<br>Cytoplasma
RPLP2	*Ribosomal protein lateral stalk subunit P2.* The encoded ribosomal phosphoprotein is a component of the 60S subunit, it belongs to the L12P family of ribosomal proteins and has a role in the elongation step of protein synthesis. The protein is located in the cytoplasm.	Ribosome 60S<br>Protein elongation<br>Cytoplasma
RPS1	*Ribosomal protein S1.*	Ribosome
RPS3	*Ribosomal protein S3*. This ribosomal protein that is a component of the 40S subunit, where it forms part of the domain where translation is initiated. It belongs to the S3P family of ribosomal proteins. Studies of the mouse and rat proteins have demonstrated that the protein has an extraribosomal role as an endonuclease involved in DNA damage. The protein appears to be located in both the cytoplasm and nucleus but not in the nucleolus. This gene is co-transcribed with the small nucleolar RNA genes U15A and U15B.	Ribosome 40S<br>Translation initiation<br>Cytoplasm, nucleus<br>Endonuclease, DNA repair
RPS5	*Ribosomal protein S5*. This gene encodes a ribosomal protein that is a component of the 40S subunit. It belongs to the S7P family of ribosomal proteins and is located in the cytoplasm.	Ribosome 40S<br>Cytoplasm
RPS8	*Ribosomal protein S8*. The protein belongs to the S8E family of ribosomal proteins and is located in the cytoplasm. This gene is co-transcribed with the small nucleolar RNA genes U38A, U38B, U39, and U40 located in its fourth, fifth, first, and second introns, respectively.	Ribosome<br>Cytoplasm

RPS9	*Ribosomal protein S9*. The encoded ribosomal protein is a component of the 40S subunit and belongs to the S4P family of ribosomal proteins. It is located in the cytoplasm.	Ribosome 40S<br>Cytoplsm
RPS17	*Ribosomal protein S17.* This gene encodes a ribosomal protein that is a component of the 40S subunit. The protein belongs to the S17E family of ribosomal proteins and is located in the cytoplasm.	Ribosome 40S<br>Cytoplasm
RPS18	*Ribosomal protein S18*. This gene encodes a ribosomal protein that is a component of the 40S subunit. The protein belongs to the S13P family of ribosomal proteins and is is located in the cytoplasm. The gene product of the E. coli ortholog (ribosomal protein S13) is involved in the binding of fMet-tRNA, and thus, in the initiation of translation.	Ribosome 40S<br>Cytoplasm<br>Translation?
RPS20	*Ribosomal protein S20*. The encoded ribosomal protein is a component of the 40S subunit. It belongs to the S10P family of ribosomal proteins and is located in the cytoplasm. This gene is co-transcribed with the small nucleolar RNA gene U54, which is located in its second intron.	Ribosome S40<br>Cytoplasm
RPS23	*Ribosomal protein S23*. This ribosomal protein is a component of the 40S subunit and belongs to the S12P family of ribosomal proteins. It is located in the cytoplasm.	Ribosome 40S<br>Cytoplasm
RPS24	*Ribosomal protein S24*. This gene encodes a ribosomal protein that is a component of the 40S subunit. The protein belongs to the S24E family of ribosomal proteins and is located in the cytoplasm.	Ribosome 40S<br>Cytoplasm
RPS28	*Ribosomal protein S28*. This gene encodes a ribosomal protein that is a component of the 40S subunit. It belongs to the S28E family of ribosomal proteins and is located in the cytoplasm.	Ribosome 40S<br>Cytoplasm
RPS29	*Ribosomal protein S29*. This gene encodes a ribosomal protein that is a component of the 40S subunit and a member of the S14P family of ribosomal proteins. It can enhance the tumor suppressor activity of Ras-related protein 1A (KREV1) and is located in the cytoplasm.	Ribosome 40S<br>Cytoplasm
SERBP1	*SERPINE1 mRNA binding protein 1.* The encoded protein enables SUMO binding activity; mRNA 3'-UTR binding activity; and ribosome binding activity. It is involved in PML body organization. The protein is located in cytosol and nucleus.	Ribosome binding<br>Cytosol and nucleus
SPL13A	*Squamosa promoter-binding protein-like (SBP domain) transcription factor family protein.*	
SRP14	*Signal recognition particle 14*. The protein has RNA binding activity and is involved in protein targeting to endoplasmic reticulum. It is also located in nucleus.	RNA binding
SRP9	*Signal recognition particle 9*. The protein is predicted to have RNA binding activity and signal recognition particle binding activity. It is also predicted to be involved in SRP-dependent cotranslational protein targeting to membrane. Predicted to be located in cytosol.	RNA binding
UTP3	*UTP3 small subunit processome component.* The protein has RNA binding activity. Predicted to be involved. It is located in the nucleolus.	RNA binding

PROTEIN STABILIZATION (4 proteins)

CCT2	*Chaperonin containing TCP1 subunit 2.* The encoded protein is a molecular chaperone that is a member of the chaperonin containing TCP1 complex (CCT), also known as the TCP1 ring complex (TRiC). Unfolded polypeptides enter the central cavity of the complex and are folded in an ATP-dependent manner. The complex folds various proteins, including actin and tubulin. The complex seems to have an antiapoptotic effect through inhibition of autophagy; inhibition of the CCT/TCPI/Alt-mTOR pathway is thereby a possible strategy for targeted therapy in human AML [74].	Molecular chaperon<br>CCT/TRiC complex<br>Actin, tubulin<br>Inhibits autophagy and apoptosis<br>AML-chemoresistance
CCT8 (see CCT2 above)	*Chaperonin containing TCP1 subunit 8.* The encoded protein is the theta subunit of the CCT chaperonin, which is abundant in cytosol and may be involved in the transport and assembly of newly synthesized proteins.	Molecular chaperon<br>CCT/ TRiC complex<br>Actin, tubulin<br>Inhibits autophagy and apoptosis<br>AML-chemoresistance
PFDN2	*Prefoldin subunit 2.* The encoded protein is a member of the prefoldin beta subunit family. It is one of six subunits of prefoldin, a molecular chaperone complex that binds and stabilizes newly synthesized polypeptides, thereby allowing them to fold correctly. The complex, consisting of two alpha and four beta subunits, forms a double beta barrel assembly with six protruding coiled-coils.	Prefoldin complex

TCP1<br>(see CCT2 above)	*t-complex 1*. The encoded protein is a molecular chaperone that is a member of the chaperonin containing TCP1 complex (CCT), also known as the TCP1 ring complex (TRiC).	Molecular chaperon<br>CCT/ TRiC complex<br>Actin, tubulin<br>Inhibits autophagy and apoptosis<br>AML-chemoresistance
	**2-DEOXYRIBONUCLEOTIDE BIOSYNTHETIC PROCESS (3 proteins)**	
CMPK2	*Cytidine/uridine monophosphate kinase 2*. This encoded oritein is an enzymes in the nucleotide synthesis salvage pathway that may participate in monocytic differentiation. The gene encodes regulatory RNAs that may be involved in carcinogens and in addition a mitochondrial protein [75,76].	Nucleotide synthesis<br>Mitochondria<br>Carcinogenesis?
DCTD	*dCMP deaminase*. The protein catalyzes the deamination of dCMP to dUMP, the nucleotide substrate for thymidylate synthase. The encoded protein is allosterically activated by dCTP and inhibited by dTTP and is found as a homohexamer. This protein uses zinc as a cofactor.	Nucleotide metabolism
RRM1	*Ribonucleotide reductase catalytic subunit M1*. This gene encodes the large and catalytic subunit of ribonucleotide reductase, an enzyme essential for the conversion of ribonucleotides into deoxyribonucleotides. A pool of available deoxyribonucleotides is important for DNA replication during S phase of the cell cycle as well as multiple DNA repair processes.	Nucleotide metabolism<br>Cell cycle<br>DNA repair

Table S6. Protein phosphorylation sites showing significantly increased levels in patients with *NPM1-Ins* compared with patients without *NPM1-Ins*. A total of 70 phosphosites were identified, and the table presents the gene name, the protein name, the position of the phosphorylated amino acid residue (position), the phosphorylated amino acid (AA) and the p-value from ANOVA analysis. The phosphosites are ranged according to their p-value. Phosphosites also identified in PPI analyses are marked with grey shadow.

Gene name	Protein name	Position	AA	ANOVA p-value
TOR1AIP1	Torsin-1A-interacting protein 1	154	S	7.52E-06
TOR1AIP1	Torsin-1A-interacting protein 1	156	S	7.52E-06
TOR1AIP1	Torsin-1A-interacting protein 1	157	S	7.52E-06
SSFA2	Sperm-specific antigen 2	739	S	3.37E-05
DTNBP1	Dysbindin	321	S	5.72E-05
DNAJC1	DnaJ homolog subfamily C member 1	479	S	7.37E-05
DNAJC1	DnaJ homolog subfamily C member 1	480	S	7.37E-05
SRP72	Signal recognition particle subunit SRP72	625	S	0.0001
PSMD1	26S proteasome non-ATPase regulatory subunit 1	315	S	0.0002
PSMD1	26S proteasome non-ATPase regulatory subunit 1	311	T	0,0002
EEPD1	Endonuclease/exonuclease/phosphatase family domain-containing protein 1	173	S	0.0002
TRPV2	Transient receptor potential cation channel subfamily V member 2	3	S	0.0002
SEC61B	Protein transport protein Sec61 subunit beta	17	S	0.0003
MTDH	Protein LYRIC	568	S	0.0004
TMEM179B	Transmembrane protein 179B	205	S	0.0004
TMEM179B	Transmembrane protein 179B	206	S	0.0004
SH3BP1	SH3 domain-binding protein 1	544	S	0.0006
PLEC	Plectin	4613	S	0.0009
DTNBP1	Dysbindin	316	S	0.0011
NUCKS1	Nuclear ubiquitous casein and cyclin-dependent kinase substrate 1	214	S	0.0018
NUCKS1	Nuclear ubiquitous casein and cyclin-dependent kinase substrate 1	204	S	0.0018
NUCKS1	Nuclear ubiquitous casein and cyclin-dependent kinase substrate 1	202	T	0.0018
FAM21A;FAM21C	WASH complex subunit FAM21A;WASH complex subunit FAM21C	619	S	0.0018
FAM21A;FAM21C	WASH complex subunit FAM21A;WASH complex subunit FAM21C	620	S	0.0018
ADAM17	Disintegrin and metalloproteinase domain-containing protein 17	791	S	0.0022
SRRM2	Serine/arginine repetitive matrix protein 2	1103	S	0.0024
BRAT1	BRCA1-associated ATM activator 1	742	S	0.0027
DPM1	Dolichol-phosphate mannosyltransferase subunit 1	9	S	0.0031
TFEB	Transcription factor EB	109	S	0.0033
TFEB	Transcription factor EB	114	S	0.0033
TFEB	Transcription factor EB	122	S	0.0033
UBE2J1	Ubiquitin-conjugating enzyme E2 J1	266	S	0.0036
MEIS1	Homeobox protein Meis1	194	S	0.0044
STX12	Syntaxin-12	142	S	0.0046
IRS2	Insulin receptor substrate 2	915	S	0.0050
IRS2	Insulin receptor substrate 2	306	S	0.0058
ADD3	Gamma-adducin	649	S	0.0062
SLC39A7	Zinc transporter SLC39A7	275	S	0.0065
SLC39A7	Zinc transporter SLC39A7	276	S	0.0065
PSMD2	26S proteasome non-ATPase regulatory subunit 2	16	S	0.0074

ZC3H14	Zinc finger CCCH domain-containing protein 14	409	S	0.0080
CCAR2	Cell cycle and apoptosis regulator protein 2	675	S	0.0104
TGOLN2	Trans-Golgi network integral membrane protein 2	298	S	0.0109
CDK12	Cyclin-dependent kinase 12	1244	T	0.0121
PLEC	Plectin	4389	S	0.0143
RCC1	Regulator of chromosome condensation	11	S	0.0146
MTDH	Protein LYRIC	426	S	0.0154
FAM21A	WASH complex subunit FAM21A	539	S	0.0156
CHAMP1	Chromosome alignment-maintaining phosphoprotein 1	282	S	0.0158
ITPR1	Inositol 1,4,5-trisphosphate receptor type 1	1716	S	0.0173
CDK12	Cyclin-dependent kinase 12	301	S	0.0177
CDK12	Cyclin-dependent kinase 12	303	S	0.0177
AFF4	AF4/FMR2 family member 4	180	S	0.0214
VPS26B	Vacuolar protein sorting-associated protein 26B	304	S	0.0215
ZEB2	Zinc finger E-box-binding homeobox 2	760	S	0.0218
ZEB2	Zinc finger E-box-binding homeobox 2	758	T	0.0218
SRRM1	Serine/arginine repetitive matrix protein 1	769	S	0.0219
BCLAF1	Bcl-2-associated transcription factor 1	339	S	0.0230
IRS2	Insulin receptor substrate 2	1149	S	0.0235
SUN2	SUN domain-containing protein 2	12	S	0.0244
ARHGAP30	Rho GTPase-activating protein 30	630	S	0.0248
PBXIP1	Pre-B-cell leukemia transcription factor-interacting protein 1	43	S	0.0272
USP20	Ubiquitin carboxyl-terminal hydrolase 20	132	S	0.0287
USP20	Ubiquitin carboxyl-terminal hydrolase 20	134	S	0.0287
LNPEP	Leucyl-cystinyl aminopeptidase	91	S	0.0301
CHD2	Chromodomain-helicase-DNA-binding protein 2	1728	S	0.032
STK11IP	Serine/threonine-protein kinase 11-interacting protein	398	S	0.0333
FCER1G	High affinity immunoglobulin epsilon receptor subunit gamma	69	S	0.0340
C16orf54	Transmembrane protein C16orf54	194	S	0.0343
CDKN2AIP	CDKN2A-interacting protein	131	S	0.0348

Table S7. The phosphoproteomic comparison of FAB-M4/M5 AML cells with and without *NPM1-Ins*; a summary of proteins forming interacting networks (**Figure 2** of the main text) and showing increased phosphorylation levels in patients with (spliceosome, proteasome) and without (ribosome biogenesis, NuRD complex) *NPM1-Ins*. The table presents the gene name, protein names and key words with regard to protein function. The table is based on information from the Gene database and selected references from the PubMed database (accessed 29th of October 2023) [77-85].

	**SPLICEOSOME (2 proteins)**	
SRRM1	*Serine and arginine repetitive matrix 1.* The protein has RNA binding activity and is predicted to be involved in mRNA splicing via spliceosomes. It is located in nuclear speck.	RNA binding<br>Spliceosome
SRRM2	*Serine and arginine repetitive matrix 2.* The encoded protein enables C2H2 zinc finger domain binding activity and protein N-terminus binding activity. The protein is involved in mRNA splicing, via effects on the spliceosome.	mRNA splicing<br>Spliceosome
	**PROTEASOME (2 proteins)**	
PSMD1	*Proteasome 26S subunit, non-ATPase 1.* The 26S proteasome is a multicatalytic proteinase complex composed of two complexes, a 20S core and a 19S regulator. The 20S core is composed of 4 rings of 28 non-identical subunits; 2 rings are composed of 7 alpha subunits and 2 rings are composed of 7 beta subunits. The 19S regulator is composed of a base, which contains 6 ATPase subunits and 2 non-ATPase subunits, and a lid, which contains up to 10 non-ATPase subunits. Proteasomes are distributed throughout eukaryotic cells at a high concentration and <u>cleave peptides</u> in an <u>ATP/ubiquitin-dependent process</u> in a non-lysosomal pathway. This gene encodes the largest <u>non-ATPase subunit</u> of the <u>19S</u> regulator lid, which is responsible for <u>substrate recognition</u> and binding. A previous study suggests that proteins within the regulatory 19S subunit (including non-ATPase subunits) can be involved in leukemogenesis in human AML [77].	19S proteasomal subunit<br>Non-ATPase<br>AML
PSMD2	*Proteasome 26S subunit ubiquitin receptor, non-ATPase 2.* This gene encodes one of the non-ATPase subunits of the 19S regulator lid. In addition to participation in proteasome function, this subunit may also participate in the Tumor necrosis factor (TNF) signaling pathway since it interacts with the TNF type 1 receptor. Furthermore, several molecules involved in or modulating Wnt signalling have a prognostic value in AML including PSMD2.	19S proteasomal subunit<br>Non-ATPase<br>TNF signaling<br>Wnt signaling<br>AML chemosensitivity
	**RIBOSOME BIOGENESIS (4 proteins)**	
UPT14A	*UTP14A small subunit processome component*. This gene encodes a member of the uridine triphosphate 14 family. It is an essential component of a large ribonucleoprotein complex <u>bound to the U3 small nucleolar RNA</u> and is involved in <u>ribosome biogenesis</u> and 18S rRNA synthesis.	Ribosome biogenesis<br>RNA binding<br>Ribonucleoprotein
KRR1	*KRR1 small subunit processome component homolog.* The encoded protein enables RNA binding activity and is predicted to be involved in rRNA processing.	tRNA processing
KRI1	*KRI1 homolog.* This protein seems to be involved in regulation of apoptosis; it can have antiapoptotic effects possibly through modulation of MPK-1/ERK signaling. It also seems to be involved in DNA repair [78].	Apoptosis?<br>DNA repair?
AATF	*Apoptosis antagonizing transcription factor.* The encoded protein was identified on the basis of its interaction with MAP3K12/DLK, a protein kinase involved in the induction of apoptosis. It contains a leucine zipper, which is a characteristic motif of transcription factors. Overexpression of this gene interfered with MAP3K12 induced apoptosis [79-82]. Due to its roles in ribosome biogenesis, chechpoint control/DNA damage and regulation of apoptosis AATF has been suggested as a possible therapeutic target in cancer treatment [83].	Ribosome biogenesis<br>Cell cycle regulation<br>Apoptosis<br>Transcription regulation?
	**NuRD COMPLEX (3 proteins)**	
SAP30	*Sin3A associated protein 30.* The encoded protein is a component of the histone deacetylase complex, which includes SIN3, SAP18, HDAC1, HDAC2, RbAp46, RbAp48 as well as other polypeptides. This complex is active in deacetylating core histone octamers, but inactive in	Histone deacetylase

	deacetylating nucleosomal histones. Targeting of Sap30 mediated signaling is regarded as a possible strategy to eradicate leukemia-initiating cells in human AML [84].	
MTA2	*Metastasis associated 1 family member 2.* This encoded protein has been identified as a component of NuRD, a nucleosome remodeling deacetylase complex. Their indirect effects on transcriptional regulation may include chromatin remodeling. It has two DNA binding domains, a dimerization domain, and a domain commonly found in proteins that methylate DNA. The protein mediates deacetylation of p53 that is correlated with loss of growth inhibition in transformed cells.	Nuclear deacetylase<br>DNA methylation?<br>p53 deacetylation
GATAD2A	*GATA zinc finger domain containing 2A.* The encoded protein enables protein-macromolecule adaptor activity. It is involved in negative regulation of transcription and is located in nucleoplasm. The protein may also interact with STAT3 to suppress its activity through posttranslational modification [85].	Transcription<br>Nucleoplasm<br>STAT3

Table S8. Protein phosphorylation sites showing significantly increased levels in patients without *NPM1-Ins* compared with patients with *NPM1-Ins*. A total of 76 phosphosites were identified, and the table presents the gene name, the protein name the position of the phosphorylated amino acid residue (position), the phosphorylated amino acid (AA)band the p-value from ANOVA analysis. The phosphosites are ranged according to their p-value. Phosphosites also identified in PPI analyses are marked with grey shadow.

Gene name	Protein name	Position	AA	ANOVA P-value
CCDC86	Coiled-coil domain-containing protein 86	18	S	3.49E-07
CCDC86	Coiled-coil domain-containing protein 86	21	S	3.49E-07
KRI1	Protein KRI1 homolog	639	S	1.27E-06
KRI1	Protein KRI1 homolog	628	S	1.39E-06
SURF6	Surfeit locus protein 6	138	S	5.71E-05
NPM1	Nucleophosmin	125	S	6.04E-05
H1FX	Histone H1x	31	S	0.0002
EZR;MSN;RDX	Ezrin;Moesin;Radixin	558	T	0.0002
SNIP1	Smad nuclear-interacting protein 1	54	S	0,0002
IQGAP2	Ras GTPase-activating-like protein IQGAP2	16	S	0.0003
SLC38A1	Sodium-coupled neutral amino acid transporter 1	52	S	0.0004
TNKS1BP1	182 kDa tankyrase-1-binding protein	1620	S	0.0004
TNKS1BP1	182 kDa tankyrase-1-binding protein	1621	S	0.0004
DDX54	ATP-dependent RNA helicase DDX54	782	S	0.0005
GATAD2A	Transcriptional repressor p66-alpha	100	S	0.0005
GATAD2A	Transcriptional repressor p66-alpha	107	S	0.0005
SNIP1	Smad nuclear-interacting protein 1	52	S	0.0005
GATAD2A	Transcriptional repressor p66-alpha	114	S	0.0006
CCDC86	Coiled-coil domain-containing protein 86	66	S	0.0008
TNKS1BP1	182 kDa tankyrase-1-binding protein	836	S	0.0009
CCDC86	Coiled-coil domain-containing protein 86	65	T	0.0009
DNTTIP1	Deoxynucleotidyltransferase terminal-interacting protein 1	161	S	0.0010
MYO18A	Unconventional myosin-XVIIIa	2041	S	0.0010
MYO18A	Unconventional myosin-XVIIIa	2043	S	0.0011
NCK1	Cytoplasmic protein NCK1	85	S	0,0018
BAZ1B	Tyrosine-protein kinase BAZ1B	158	S	0.0019
UHRF1	E3 ubiquitin-protein ligase UHRF1	287	S	0.0020
HIST1H1B	Histone H1.5	18	S	0.0020
SKAP2	Src kinase-associated phosphoprotein 2	283	S	0.0022
CCDC86	Coiled-coil domain-containing protein 86	69	S	0.0023
FTSJ3	pre-rRNA processing protein FTSJ3	336	S	0.0024
CHAMP1	Chromosome alignment-maintaining phosphoprotein 1	87	S	0.0024
HIST1H1D	Histone H1.3	18	T	0.0029
FTSJ3	pre-rRNA processing protein FTSJ3	335	S	0.0029
NAP1L4	Nucleosome assembly protein 1-like 4	125	S	0.0030
SCAF11	Protein SCAF11	481	S	0.0032
SCAF11	Protein SCAF11	648	S	0.0032
RBM25	RNA-binding protein 25	677	S	0.0038
MTA2	Metastasis-associated protein MTA2	435	S	0.0042
NMT1	Glycylpeptide N-tetradecanoyltransferase 1	47	S	0.0051

MYO18A	Unconventional myosin-XVIIIa	1998	S	0.0052
MYO18A	Unconventional myosin-XVIIIa	2002	S	0.0052
HMGA1	High mobility group protein HMG-I/HMG-Y	103	S	0.0054
RBM39	RNA-binding protein 39	117	S	0.0057
SMARCC1	SWI/SNF complex subunit SMARCC1	328	S	0.0058
SMARCC1	SWI/SNF complex subunit SMARCC1	330	S	0.0058
DDX21	Nucleolar RNA helicase 2	121	S	0.0060
HMGA1	High mobility group protein HMG-I/HMG-Y	102	S	0.0063
PPAN	Suppressor of SWI4 1 homolog	238	S	0.0070
FNBP4	Formin-binding protein 4	499	S	0.0071
FNBP4	Formin-binding protein 4	508	S	0.0071
AATF	Protein AATF	316	S	0.0077
AATF	Protein AATF	320	S	0.0077
AATF	Protein AATF	321	S	0.0077
SNIP1	Smad nuclear-interacting protein 1	58	S	0.0092
UTP14A	U3 small nucleolar RNA-associated protein 14 homolog A	29	S	0.0093
UTP14A	U3 small nucleolar RNA-associated protein 14 homolog A	31	S	0.0093
SAP30	Histone deacetylase complex subunit SAP30	131	S	0.0093
SAP30	Histone deacetylase complex subunit SAP30	138	S	0.0097
TNKS1BP1	182 kDa tankyrase-1-binding protein	1666	S	0.0100
DDX54	ATP-dependent RNA helicase DDX54	39	S	0.0119
NCOR1	Nuclear receptor corepressor 1	172	S	0.0130
DDX54	ATP-dependent RNA helicase DDX54	41	S	0.0152
ACACA	Acetyl-CoA carboxylase 1; Biotin carboxylase	80	S	0.0155
KRR1	KRR1 small subunit processome component homolog	3	S	0.0157
TFAM	Transcription factor A, mitochondrial	193	S	0.0159
EIF5B	Eukaryotic translation initiation factor 5B	214	S	0.0166
TMPO	Lamina-associated polypeptide 2, isoforms beta/gamma	385	S	0.0174
SMARCAD1	SWI/SNF-related matrix-associated actin-dependent regulator of chromatin subfamily A containing DEAD/H box 1	212	S	0.0177
PA2G4	Proliferation-associated protein 2G4	2	S	0.0238
TMPO	Lamina-associated polypeptide 2, isoform alpha	160	T	0.0246
MKI67	Antigen KI-67	2528	S	0.0252
DOCK2	Dedicator of cytokinesis protein 2	1705	S	0.0293
EIF5B	Eukaryotic translation initiation factor 5B	135	S	0.0297
EIF5B	Eukaryotic translation initiation factor 5B	137	S	0.0297
PDCD5	Programmed cell death protein 5	119	S	0.0308

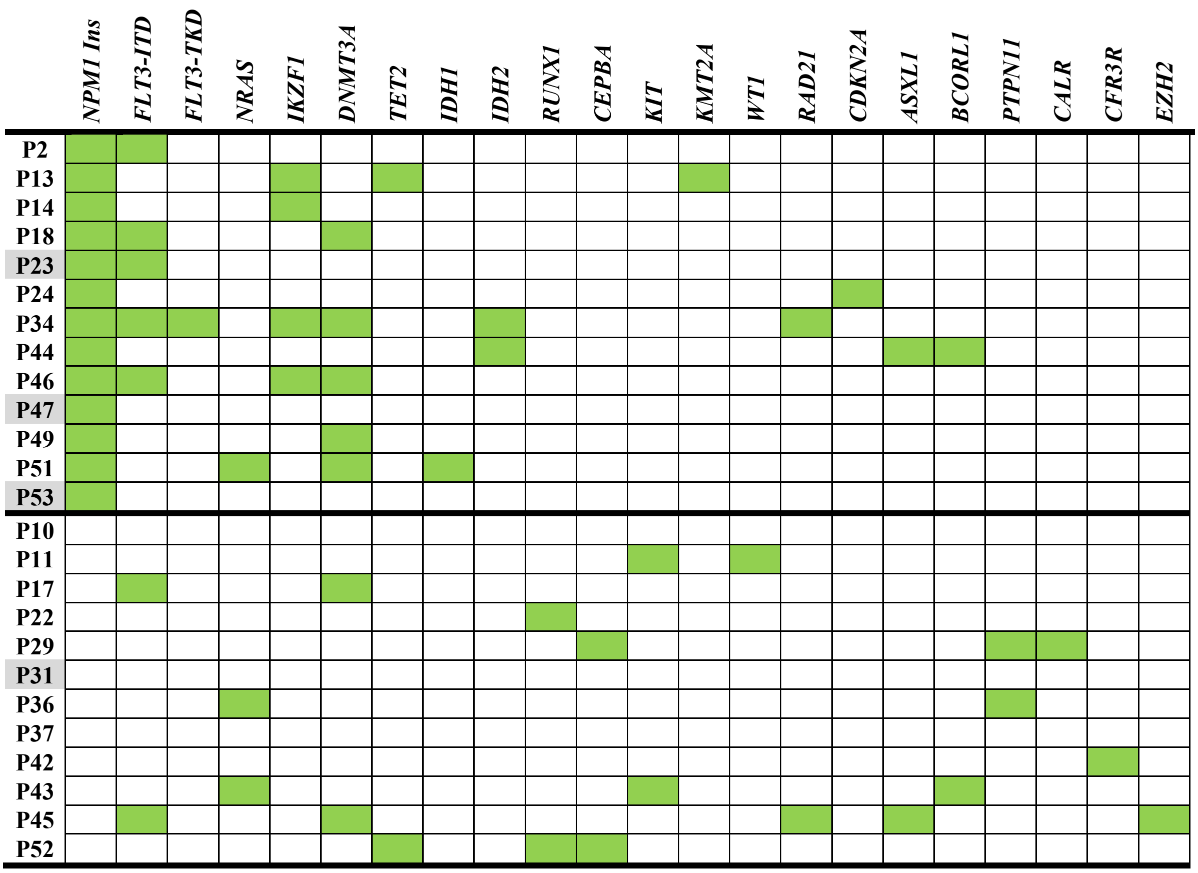

Figure S1. Molecular genetic analyses of primary AML cells with (top rows) and without (bottom rows) *NPM1-Ins*. We performed a submicroscopic mutational profiling of 54 genes frequently mutated in AML by characterizing the Illuminas TruSight Myeloid Gene Panel [127]. The mutated genes are indicated at the top of the figure, the patient identity is presented in the left column and listed in the same way as in **Table S1**. The figure presents the results only for those genes that were mutated for at least one of the patients included in the present study. The four patients marked with grey were only tested for *NPM1* and *FLT3* abnormalities.

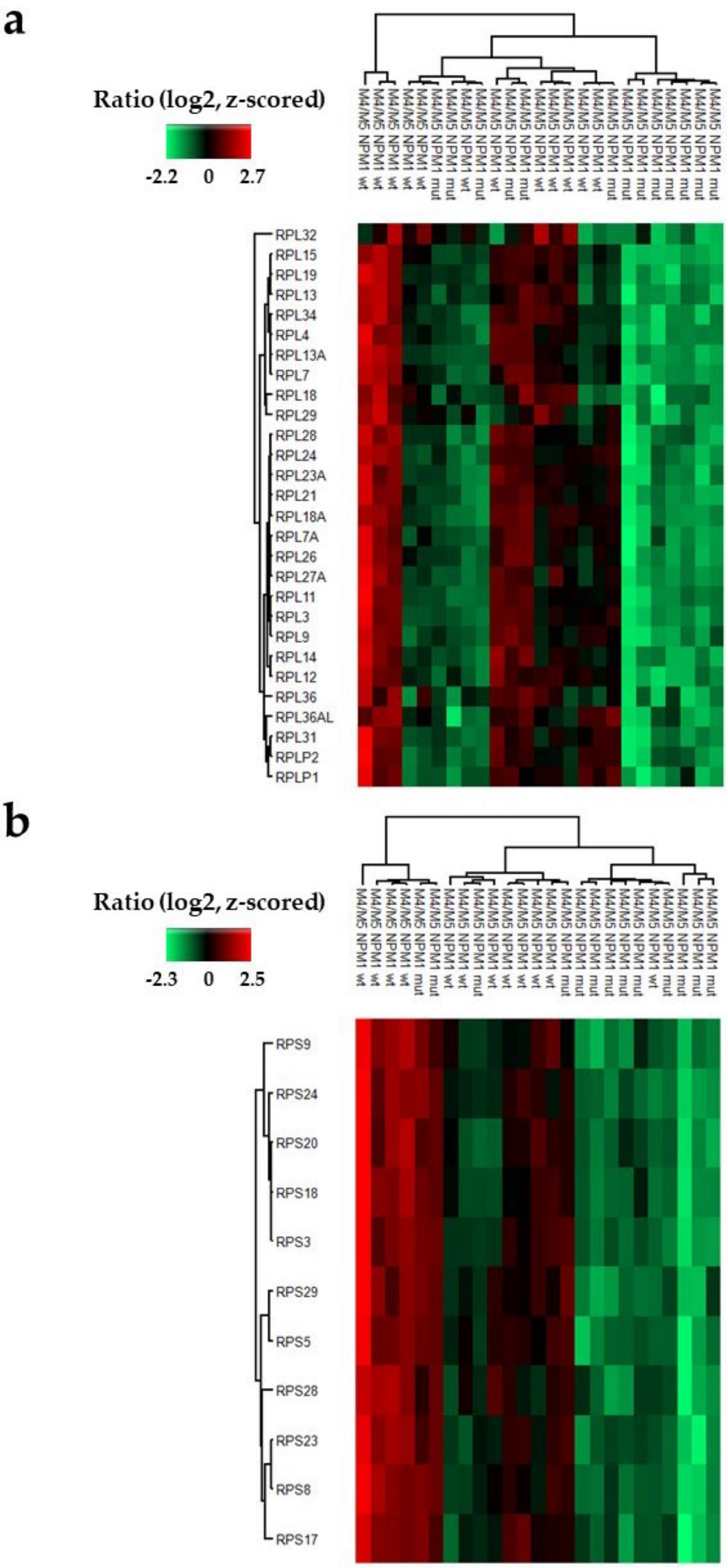

Figure S2. Hierarchical clustering analysis including all our FAB-M4/M5 patients and based on ribosomal proteins included in identified protein-protein interaction (PPI) networks. The two analyses were based on the differentially expressed large (**a**) and small (**b**) ribosomal subunit proteins with a significantly different regulation identified by ANOVA. Samples with and without *NPM1-Ins* are referred to as *NPM1* mut and *NPM1* wt, respectively, in this figure.

a

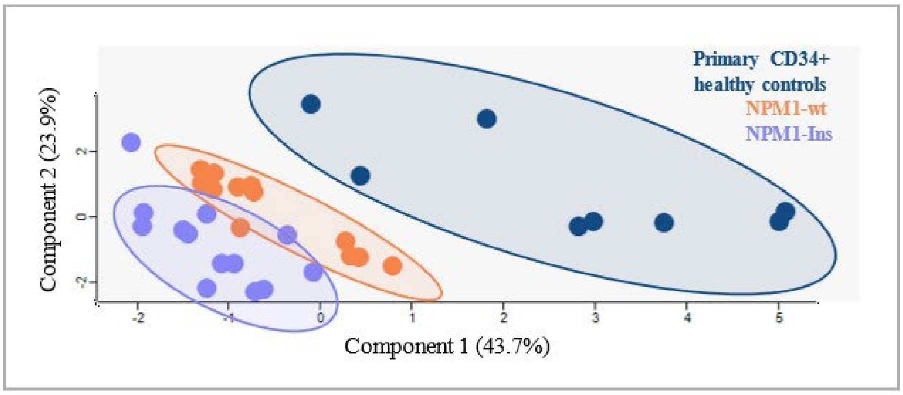

b

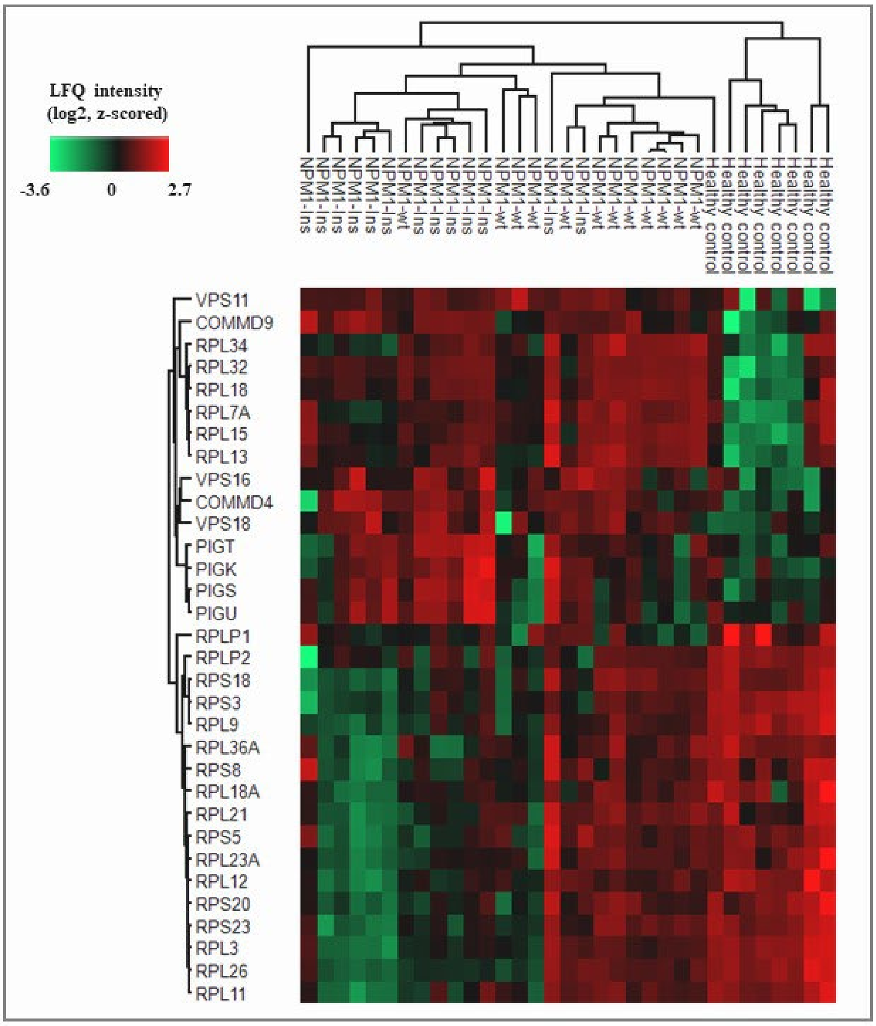

Figure S3. Principal component (**a**) and unsupervised hierarchical clustering (**b**) analysis of FAB-M4/M5 patients with and without (referred to as *NPM1-wt* in the figure) *NPM1-Ins;* a comparison with normal $CD34^+$ bone marrow cells (referred to as healthy controls in the figure). The label-free quantification (LFQ) analysis was based on the expression of the 32 out of 53 differentially expressed proteins that were identified by analysis of PPIs in **Figures 1b and 1d** after ANOVA test with quantitative values in all $CD34^+$ as well as in patient samples.

a

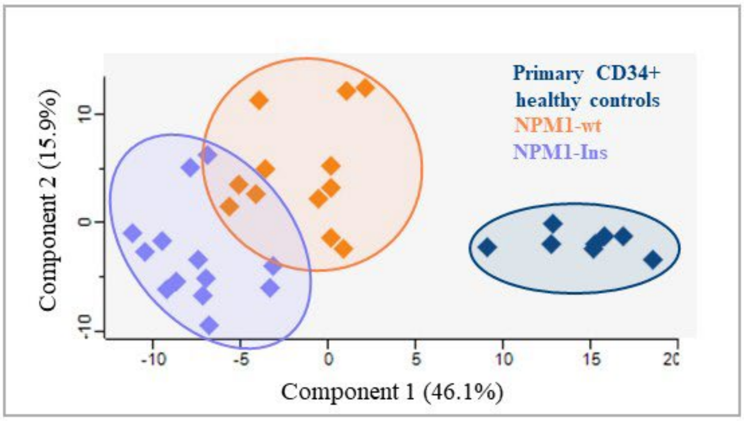

b

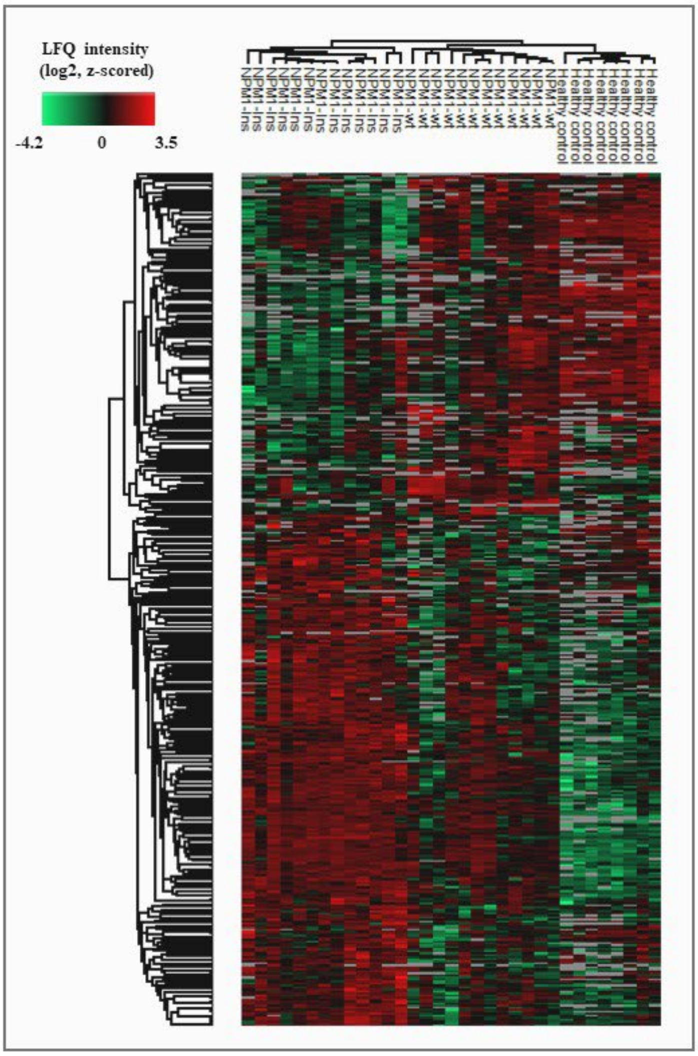

Figure S4. Principal component (**a**) and unsupervised hierarchical clustering (**b**) analysis of FAB-M4/M5 patients with and without (referred to as *NPM1-wt* in the figure) *NPM1-Ins;* a comparison with normal $CD34^+$ bone marrow cells (referred to as healthy controls in the figure). The LFQ analysis was based on the expression of 426 differentially regulated proteins found after a *t*-test perfomance.
